# Immunometabolic Dysregulation in B-Cell Acute Lymphoblastic Leukemia Revealed by Single-Cell RNA Sequencing: Perspectives on Subtypes and Potential Therapeutic Targets

**DOI:** 10.3390/ijms26209996

**Published:** 2025-10-14

**Authors:** Dingya Sun, Dun Hu, Jialu Wang, Jun Peng, Shan Wang

**Affiliations:** 1 Xiangya School of Pharmaceutical Sciences, Department of Pharmacology, Central South University, Changsha 410083, China; sundingya627@163.com; 2 Department of Pharmaceutical Engineering, College of Chemistry and Chemical Engineering, Central South University, Changsha 410083, China; huduncsu@163.com (D.H.); m16680808529@163.com (J.W.)

**Keywords:** B-cell acute lymphoblastic leukemia, scRNA-seq, bulk RNA-seq, immunometabolism, monocyte, T cells, machine learning model

## Abstract

B-cell acute lymphoblastic leukemia (B-ALL) is characterized by the abnormal proliferation of B-lineage lymphocytes in the bone marrow (BM). The roles of immune cells within the BM microenvironment remain incompletely understood. Single-cell RNA sequencing (scRNA-seq) provides the potential for groundbreaking insights into the pathogenesis of B-ALL. In this study, scRNA-seq was conducted on BM samples from 17 B-ALL patients (B-ALL cohorts) and 13 healthy controls (HCs). Bioinformatics analyses, including clustering, differential expression, pathway analysis, and gene set variation analysis, systematically identified immune cell types and assessed T-cell prognostic and metabolic heterogeneity. A metabolic-feature-based machine learning model was developed for B-ALL subtyping. Furthermore, T-cell–monocyte interactions, transcription factor (TF) activity, and drug enrichment analyses were performed to identify therapeutic targets. The results indicated significant increases in Pro-B cells, alongside decreases in B cells, NK cells, monocytes, and plasmacytoid dendritic cells (pDCs) among B-ALL patients, suggesting immune dysfunction. Clinical prognosis correlated significantly with the distribution of T-cell subsets. Metabolic heterogeneity categorized patients into four distinct groups (A–D), all exhibiting enhanced major histocompatibility class I (MHC-I)-mediated intercellular communication. The metabolic-based machine learning model achieved precise classification of B-ALL groups. Analysis of TF activity underscored the critical roles of MYC, STAT3, and TCF7 within the B-ALL immunometabolic network. Drug targeting studies revealed that dorlimomab aritox and palbociclib specifically target dysregulation in ribosomal and CDK4/6 pathways, offering novel therapeutic avenues. This study elucidates immunometabolic dysregulation in B-ALL, characterized by altered cellular composition, metabolic disturbances, and abnormal cellular interactions. Key TFs were identified, and targeted drug profiles were established, demonstrating the significant clinical potential of integrating immunological mechanisms with metabolic regulation for the treatment of B-ALL.

## 1. Introduction

B-ALL is a highly aggressive malignant hematologic disorder characterized by the abnormal proliferation of B-lineage lymphocytes in the bone marrow (BM) [1]. These malignant cells not only infiltrate the BM cavity, disrupting normal hematopoiesis and resulting in the significant presence of blasts and immature lymphocytes in the peripheral blood, but they also exhibit self-renewal capabilities [1,2]. The pathogenesis of B-ALL involves the aberrant activation of multiple molecular pathways, dysregulation of cellular control mechanisms, and evasion of apoptosis through various means, ultimately driving disease progression [2,3].

The pathological mechanism of B-ALL encompasses not only metabolic abnormalities but also a significant dysfunction of the immune system [4,5]. One of the defining characteristics of B-ALL is the alteration in the population of immune cells within the BM, which is specifically manifested as dual abnormalities in both the quantity and function of immune cells, including T lymphocytes and monocytes. There are notable changes in the proportional distribution of these cells, accompanied by dysregulation of their functional states [6,7]. These abnormal immune cells actively participate in and drive the pathophysiological processes associated with B-ALL. Furthermore, the immune cell populations in patients with B-ALL exhibit considerable heterogeneity, which is evident in both the distribution of various subsets and the dysfunction of their biological activities, collectively exacerbating the imbalance within the immune system [5,8].

In the pathogenesis of B-ALL, T cells and monocytes play pivotal roles [6,7]. Research indicates that T cell exhaustion occurring during diagnosis and treatment is a critical factor contributing to disease progression. Patients’ T cells not only generally exhibit an activated state but also include a subset characterized by exhaustion, marked by the expression of inhibitory receptors, which display significant heterogeneity [9]. These immunological features are closely associated with disease progression and responses to immunotherapy [10]. Meanwhile, the proportion of monocytes in the BM of B-ALL patients is significantly lower than the normal range, likely due to the suppression of normal hematopoiesis—particularly the generation of the monocyte lineage—resulting from the excessive proliferation of leukemia cells [5,6]. More importantly, the abnormalities in monocytes not only reflect the disease state but also contribute to disease progression and influence treatment prognosis [5,6]. However, the molecular mechanisms underlying these immune cells in B-ALL, particularly the heterogeneity of T cells and monocytes, the differentiation of functional subsets, and their dynamic interactions within the microenvironment at the single-cell level, have not yet been fully elucidated and remain largely enigmatic.

**Figure 1:**
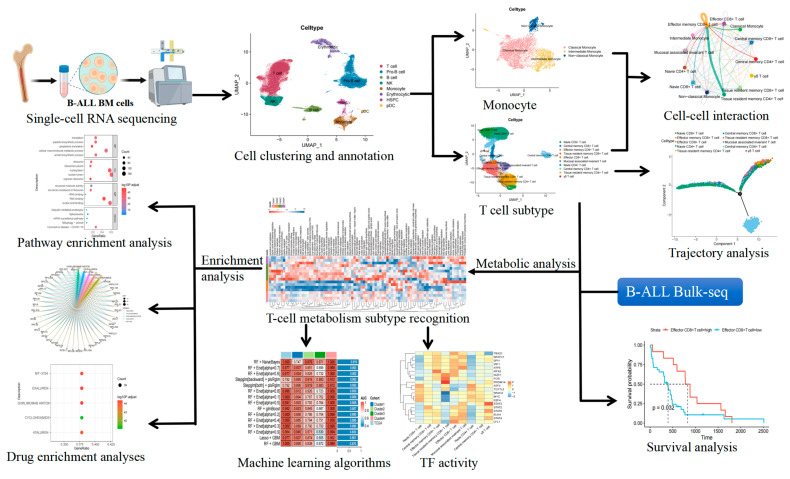
Workflow of the present study. B-cell acute lymphoblastic leukemia (B-ALL), bone marrow (BM), and transcription factor (TF).

The advent of scRNA-seq technology has enabled precise analysis of tissue heterogeneity at the cellular level, allowing for systematic characterization of distinct cell populations and their functional states within complex biological samples [11]. In the context of B-ALL, this technology serves as a vital tool for an in-depth examination of the BM immune microenvironment. By systematically analyzing gene expression patterns at the single-cell level, this method not only accurately identifies various cell subtypes but also opens new avenues for investigating the molecular mechanisms underlying disease progression. This study systematically examined the characteristics of the BM immune microenvironment in patients with B-ALL by integrating scRNA-seq and bulk RNA sequencing (bulk RNA-seq) data. Previous research and our preliminary analysis have indicated that monocytes undergo significant proportional changes in B-ALL, and their reprogramming of functional states is a crucial factor driving immunosuppression and disease progression [5,6]. Therefore, we specifically focus on the dynamic interactions between T cells and monocytes to uncover the core mechanisms underlying the dysregulation of the immune microenvironment in B-ALL. As illustrated in Figure 1, the entire research process comprehensively outlines the analytical procedure, providing a substantial theoretical foundation for the development of novel therapeutic strategies for B-ALL.

## 2. Results

### 2.1. Cellular Composition of Bone Marrow Samples

**Figure 2:**
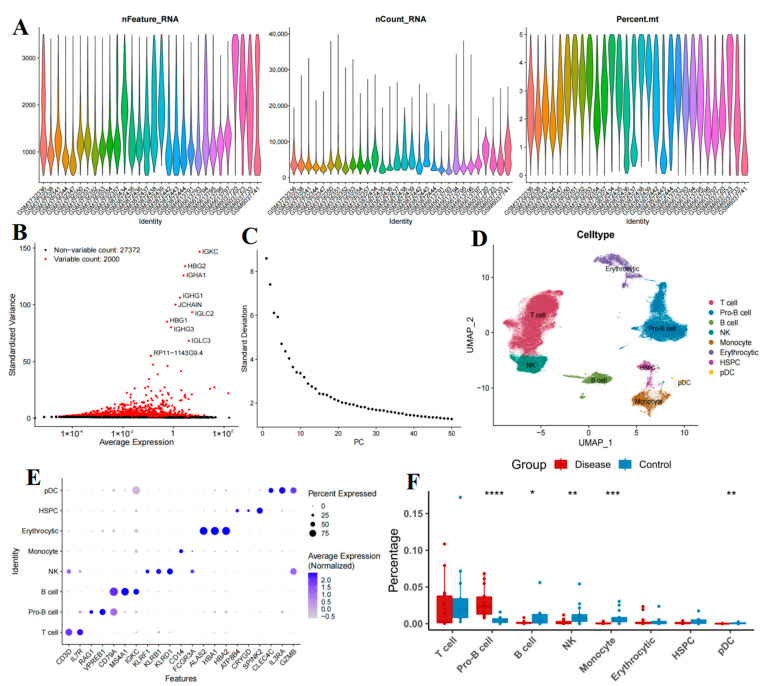
Integration and clustering of B-ALL scRNA-Seq data. (**A**) Violin plots displaying key quality metrics for each sample before and after rigorous quality control (QC). The plots show the distribution of counts per cell (nCount_RNA), features per cell (nFeature_RNA), and the percentage of mitochondrial gene expression (percent.mt). Cells with nFeature_RNA < 500 or >3500, or percent.mt > 5% were filtered out. This process ensured that only high-quality cells were retained for downstream analysis. (**B**) Volcano plot of differentially expressed genes (DEGs) identified between the B-ALL and HC cohorts after integration and batch correction. The black dots represent no highly variable genes, while the red dots denote the top 2000 highly variable genes selected. (**C**) Principal component number selection plot. For determining the optimal number of principal components (PCs) for downstream clustering analysis. The plot visualizes the standard deviation of each PC. (**D**) Eight major immune cell type annotations. (**E**) Expression of marker genes in different cell types. (**F**) The proportions of eight immune cell types in the B-ALL group and the healthy control group. *p*-values are indicated as follows: * *p* ≤ 0.05, ** *p* ≤ 0.01, *** *p* ≤ 0.001, and **** *p* ≤ 0.0001.

To ensure the reliability of the analysis data, we implemented stringent quality control measures on the raw data (Figure 2A–C). Clustering analysis revealed 20 distinct cell clusters. By identifying DEGs within these clusters and annotating them based on established marker genes (Supplementary Table S1 and Figure S1), we classified the cells into eight types: T cells, Pro-B cells, B cells, natural killer (NK) cells, monocytes, erythrocytes, hematopoietic stem and progenitor cells (HSPCs), and plasmacytoid dendritic cells (pDCs) (Figure 2D). Subsequently, we validated the annotation results by visualizing the expression of classical cell marker genes across various cell clusters (Figure 2E). As illustrated in the figure, well-established marker genes (e.g., CD3D for T cells, CD79A for B cells, etc.) demonstrated specific high expression in their respective cell types, thereby confirming the accuracy of our cell annotations. The differences in cell type proportions between the B-ALL cohorts and the healthy control (HC) cohorts were evaluated using the Wilcoxon test. The results indicated a significant increase in the proportion of Pro-B cells in the B-ALL cohorts (*p* < 0.0001), whereas the proportions of B cells (*p* < 0.05), NK cells (*p* < 0.01), monocytes (*p* < 0.001), and pDCs (*p* < 0.01) were significantly elevated in the HC cohorts. No significant differences were observed in T cells, erythrocytes, or HSPCs (Figure 2F).

### 2.2. Altered Proportions of T Cell Subtypes in Patients with B-Cell Acute Lymphoblastic Leukemia

**Figure 3:**
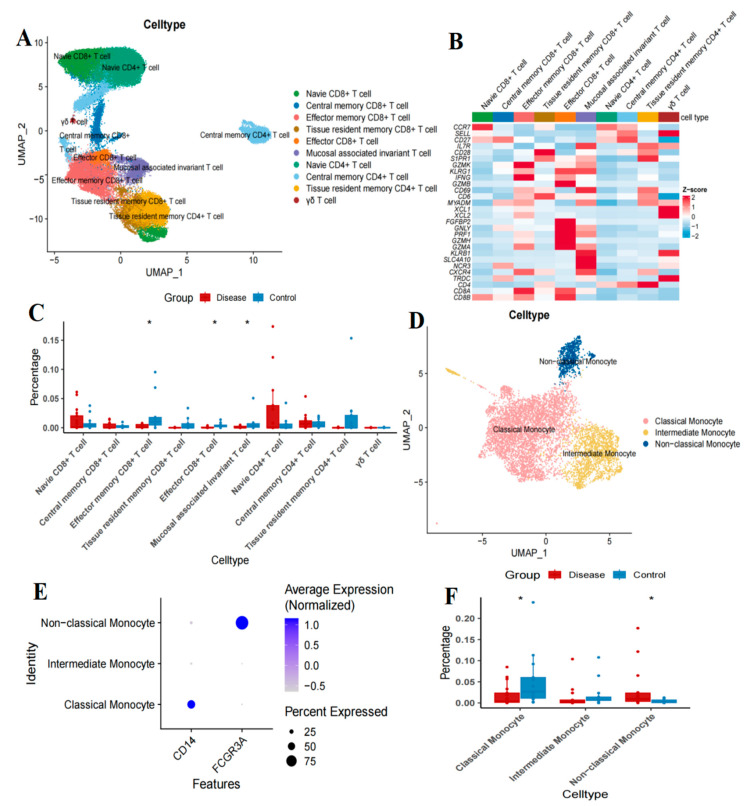
Analysis of T cells and monocytes in the B-ALL and HC cohorts. (**A**) T cell subsets annotations. (**B**) Heatmap of marker gene expression across different T cell subsets. (**C**) The proportion of different T cell subsets in the B-ALL cohorts and the HC cohorts. (**D**) Monocyte subsets analysis. (**E**) Bubble plot of marker gene expression in monocyte subsets. (**F**) Compare the proportions of three monocyte subsets. *p*-values are indicated as follows: * *p* ≤ 0.05.

Based on the gene expression profiles, we conducted dimensionality reduction and clustering analysis on T cells, identifying a total of ten distinct subsets. These subtypes include Naive CD8^+^ T (CD8^+^ TN) cells, central memory CD8^+^ T (CD8^+^ TCM) cells, effector memory CD8^+^ T (CD8^+^ TEM) cells, tissue-resident memory CD8^+^ T (CD8^+^ TRM) cells, effector CD8^+^ T (CD8^+^ TEFF) cells, mucosal-associated invariant T (MAIT) cells, naive CD4^+^ T (CD4^+^ TN) cells, central memory CD4^+^ T (CD4^+^ TCM) cells, tissue-resident memory CD4^+^ T (CD4^+^ TRM) cells, and γδ T cells (Figure 3A). Subsequently, we analyzed the expression of cell type marker genes across different T cell subsets (Figure 3B). The Wilcoxon test was employed to assess the differences in T cell subset proportions between the B-ALL cohorts and the HC cohorts. The results demonstrated that, compared to the HC cohorts, the proportions of CD8^+^ TEM, CD8^+^ TEFF, and MAIT cells were significantly reduced in the B-ALL cohorts (*p* < 0.05) (Figure 3C). No significant differences were observed in CD8^+^ TN, CD8^+^ TCM, CD8^+^ TRM, CD4^+^ TN, CD4^+^ TCM, CD4^+^ TRM, and γδ T cells (Figure 3C).

### 2.3. Trajectory of T-Cell Maturation in B-ALL Bone Marrow

**Figure 4:**
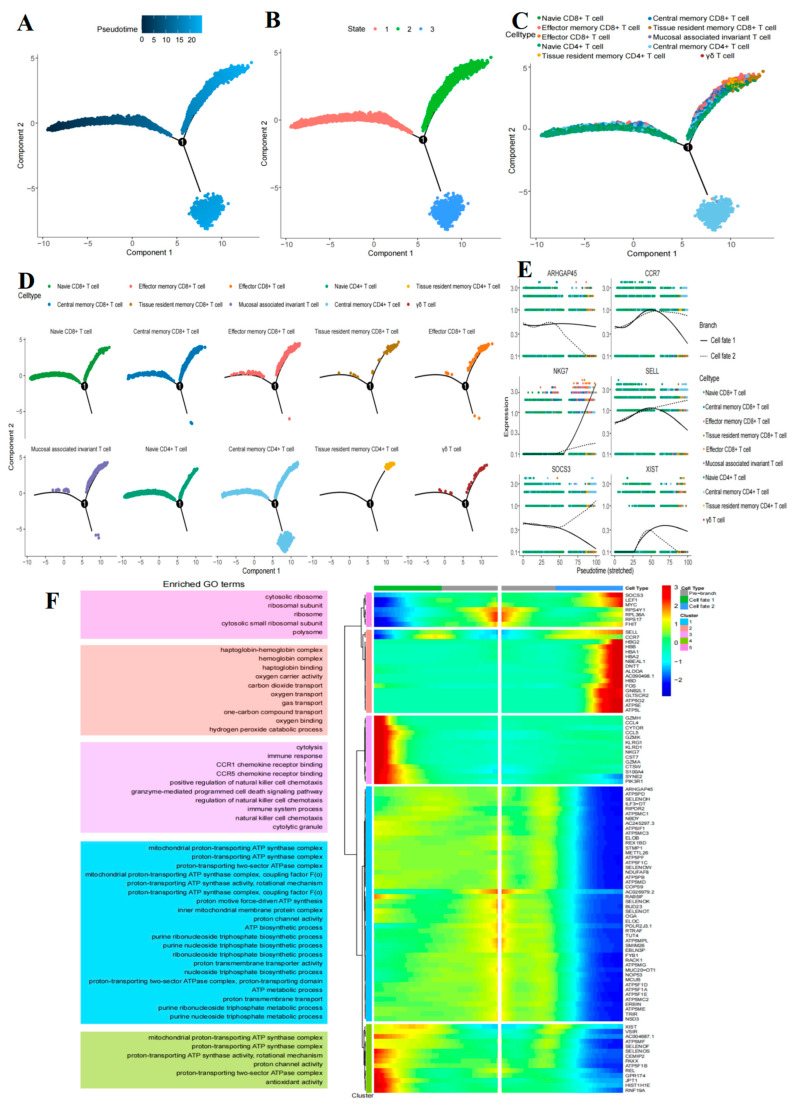
Trajectory analysis of T cells in B-ALL. (**A**) T cell subsets differentiation and development timeline (colors from dark to light represent the order of pseudotime). (**B**) Distribution of different states in pseudotime. (**C**) Distribution map of T cell subsets in pseudotime. (**D**) Separate display—Distribution map of T cell subsets in pseudotime. (**E**) The relative expression of key genes along the pseudotime trajectory (the x-axis represents pseudotime, and the y-axis represents gene expression levels). (**F**) Heatmap of differentiation of two lineages at the same time point for key marker genes and the top GO terms for each taxon (the horizontal axis of the heatmap represents the pseudotime points, while the vertical axis denotes the genes. The center of the heatmap indicates the initiation of pseudotime, with the right side of the center illustrating one lineage of pseudotime and the left side illustrating another lineage. ‘Pre-branch’ corresponds to State 1; ‘Cell fate 1’ corresponds to State 2; and ‘Cell fate 2’ corresponds to State 3. The color gradient transitioning from blue to red in the heatmap reflects the varying levels of gene expression.

To explore the global functional continuum of T cells transitioning from the naive state to the effector state, we conducted a pseudotime analysis on all T cells using Monocle2. The results of this analysis categorized the cell states into ten distinct groups (Figure 4A–C) and revealed a prominent differentiation pathway. The initial point of the trajectory (State 1) primarily comprised CD4^+^ TN cells and CD8^+^ TN cells, which exhibited similar transcriptional characteristics, reflecting their shared activated and quiescent states. Subsequently, the cell fate diverges into two major branches, with the State 2 branch representing a developmental pathway for effector, cytotoxic, and tissue-resident T cells. The terminus of this branch is predominantly enriched with terminally differentiated CD8^+^ T cell subsets, including CD8^+^ TRM cells, effector CD8^+^ T cells, and innate-like T cells such as MAIT and γδ T cells. Additionally, a subset of CD4^+^ TRM cells is also present within this branch, indicating that in the pathological context of B-ALL, they share partial benefits and transcriptional programs related to tissue residency with CD8^+^ TRM cells, despite their different lineages. The State 3 branch represents a relatively independent differentiation pathway, predominantly composed of CD4^+^ TCM cells. This suggests that this subset may be regulated by unique signaling pathways, with a developmental trajectory distinct from that of terminal effector cells. It is important to emphasize that the trajectory constructed through pseudotime analysis is based on the arrangement of cells according to gene expression similarity, reflecting a continuum of cell states (such as from naive to activated, and subsequently to effector or exhausted), rather than strict lineage differentiation relationships. Therefore, the trajectory map reveals not a lineage conversion from CD4^+^ to CD8^+^, but rather the functional state reprogramming collectively experienced by T cells of different origins under the microenvironmental pressure of B-ALL, ultimately converging to similar effector or exhausted states (Figure 4A–D).

To gain a deeper understanding of the various modules controlling gene expression during the initial differentiation of T cell subsets, we conducted a hierarchical clustering analysis of key genes using Monocle2 software. From these genes, we selected representative genes based on their significance and known biological functions. These include the cytotoxic gene *NKG7*, the migration and naive state genes *CCR7* and *SELL*, the signaling regulatory gene *SOCS3*, the metabolism-related gene *ARHGAP45*, and the X chromosome inactivation gene X-inactive specific transcript (XIST). We constructed a pseudo-time heatmap to illustrate their dynamic expression trends across different fate branches (Branch 1 and Branch 2) (Figure 4E). The results indicated that suppressor of cytokine signaling 3 (SOCS3) and lymphoid enhancer binding factor 1 (LEF1) exhibited low expression levels at the early stages of the pseudotime trajectory at this node. However, their expression gradually increased along the Cell Fate 2 branch as pseudotime progressed. In contrast, genes such as granzyme H (*GZMH*) and natural killer cell granule protein 7 (*NKG7*) demonstrated a consistent increase in expression along the Cell Fate 1 branch. Additionally, we observed that mitochondrial-related genes, including *ARHGAP45* (also known as histocompatibility antigen 1 (*HMHA1*)) and O-GlcNAcase (*OGA*), exhibited a gradual decrease in expression along the Cell Fate 2 branch as pseudotime increased (Figure 4E) (Supplementary Table S2). Subsequent GO enrichment analysis of gene modules across different clusters revealed that genes such as *ARHGAP45* and ATP synthase subunit d (*ATP5PD*) in Cluster 1 were significantly enriched in functions related to the mitochondrial proton-transporting ATP synthase complex (Figure 4F) (Supplementary Table S2). Figure 4E illustrates the dynamic expression patterns of six key genes throughout differentiation. For instance, the *ARHGAP45* gene maintains a steady expression on the Cell Fate 1 branch as pseudotime increases, while its expression gradually declines on the Cell Fate 2 branch. Conversely, the natural killer cell granule protein 7 (*NKG7*) gene exhibits stable expression on the Cell Fate 2 branch with increasing pseudotime, whereas its expression level rises on the Cell Fate 1 branch. Additionally, the selectin L (*SELL*) and suppressor of cytokine signaling 3 (*SOCS3*) genes show a decrease in expression on the Cell Fate 1 branch as pseudotime progresses, while their expression increases on the Cell Fate 2 branch (Figure 4E). The trajectory reveals potential pathways for T-cell state transformation in the context of B-ALL disease. Its branching patterns and gene expression dynamics suggest possible differences from differentiation under normal physiological conditions, which will be discussed in detail later.

### 2.4. Prognostic Value of T Cell Subsets for B-Cell Acute Lymphoblastic Leukemia

**Figure 5:**
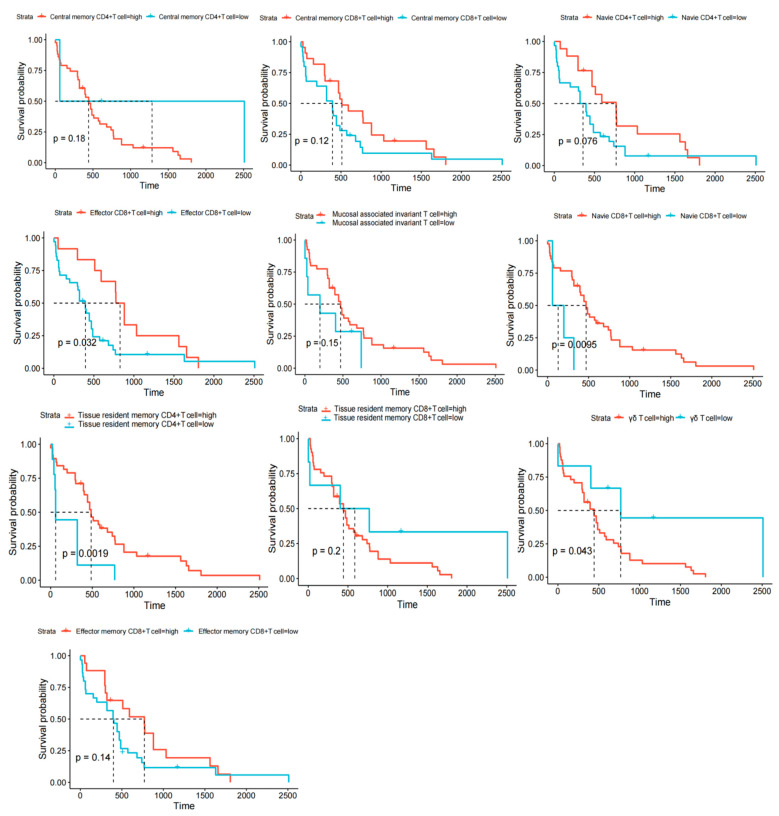
Kaplan–Meier (KM) analysis of B-ALL cohorts based on ten T cell subsets. The KM survival curve demonstrates the difference in survival rates between the “high” and “low” groups of patients based on the enrichment scores of T-cell subset marker gene sets.

To investigate the prognostic significance of various T-cell subsets in B-ALL, transcriptomic data were analyzed and quantified using Gene Set Variation Analysis based on T-cell subset-specific marker gene sets (Supplementary Table S3). Subsequently, the association between different T-cell subsets and the clinical prognosis of B-ALL patients was evaluated through KM survival analysis. The results indicated that CD8^+^ TN, CD8^+^ TEFF, CD4^+^ TRM, and γδ T cells were significantly associated with patient prognosis (*p* < 0.05) (Figure 5).

### 2.5. Changes in Monocyte Subsets in Patients with B-Cell Acute Lymphoblastic Leukemia

Considering the close association of monocytes and T cells with the progression of B-ALL [5,6,7]. A subsequent in-depth analysis of monocytes identified three distinct subsets: classical monocytes (CD14^+^), non-classical monocytes (CD16^+^), and intermediate monocytes (CD14^+^, CD16^+^), as illustrated in Figure 3D. Following this, we analyzed the expression patterns of marker genes across these subsets, as shown in Figure 3E. The differences in the proportions of monocyte subsets between the B-ALL cohorts and the HC cohorts were evaluated using the Wilcoxon test. The results indicated that, in comparison to the HC cohorts, the proportion of classical monocytes in the B-ALL group was significantly reduced (*p* < 0.05), whereas the proportion of non-classical monocytes was significantly elevated (*p* < 0.05) (Figure 3F).

### 2.6. Metabolic Heterogeneity of T Cell Subsets in B-Cell Acute Lymphoblastic Leukemia

**Figure 6:**
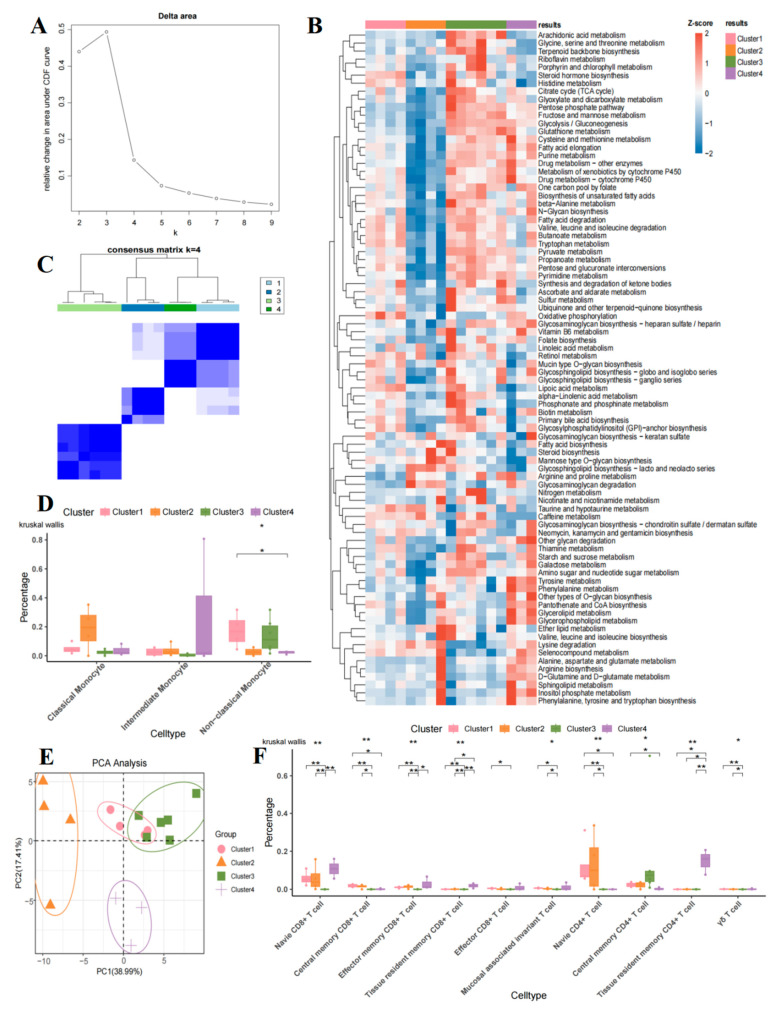
Metabolic heterogeneity and immunological differences in T cell subsets of B-ALL. (**A**) Select the optimal k value based on the delta area plot for k values ranging from 2 to 9. (**B**) Consistency clustering heatmap of metabolic pathway scores. (**C**) Clustering heatmap of expression levels of 85 metabolic pathways in four clusters (Group A–D). (**D**) Box plot of the proportions of ten T cell subsets in the four clustering groups (Group A–D). (**E**) The PCA plot represents four groups. (**F**) Box plot of the proportions of three monocyte subsets in the four clustering groups. Different B-ALL clusters are labeled using “Group” (e.g., Group A, Group B, etc.). *p*-values are indicated as follows: * *p* ≤ 0.05, ** *p* ≤ 0.01.

To investigate the metabolic heterogeneity of T cell subsets in B-ALL, we employed the scMetabolism algorithm to score 85 metabolism-related pathways from the KEGG database for individual cells within each subset (Supplementary Table S4). Unsupervised consensus clustering was performed based on the average scores of metabolic pathways across samples, with the optimal number of clusters (k = 4) determined using the incremental area value domain elbow method. Consequently, the samples were categorized into four distinct groups (Figure 6A). The clustering heatmap visually illustrates the grouping characteristics of the samples, designated as Cluster 1 to Cluster 4 (group A–D) (Figure 6C). Additionally, the results of the PCA further validated the clustering pattern observed (Figure 6E). Specifically, group A comprises 4 cases, group B comprises 4 cases, group C comprises 6 cases, and group D comprises 3 cases of B-ALL cohorts (Figure 6E) (Supplementary Table S4).

Metabolic function enrichment analysis revealed significant specificity in metabolic pathways across the various clusters (Figure 6B). Group A showed notable enrichment in metabolic profiles, including steroid hormone biosynthesis, histidine metabolism, fatty acid degradation, valine, leucine and isoleucine degradation, butanoate metabolism, tryptophan metabolism, oxidative phosphorylation, retinol metabolism, mucin type O-glycan biosynthesis, lipoic acid metabolism, taurine and hypotaurine metabolism, caffeine metabolism, lysine degradation, and selenocompound metabolism. The characteristics of active metabolism related to amino acid degradation (specifically histidine, tryptophan, branched-chain amino acids, and lysine), fatty acid degradation, and oxidative phosphorylation indicate a reliance on catabolic processes. This observation likely reflects the functional adaptive state of T cells, which sustain their survival through catabolism in a nutrient-deprived microenvironment.

Group B demonstrated substantial enrichment in metabolic profiles, including fatty acid biosynthesis, steroid biosynthesis, mannose type O-glycan biosynthesis, arginine and proline metabolism, glycosaminoglycan degradation, lysine degradation and selenocompound metabolism. This phenomenon is characterized by active metabolic pathways, including de novo fatty acid synthesis, glycosphingolipid biosynthesis, and the coordinated biosynthesis of steroids and O-glycans. In quiescent T cells, active fatty acid synthesis does not necessarily facilitate proliferation; rather, it may function in ‘metabolic preprogramming’ and energy storage—for instance, by contributing to the synthesis of cell membranes and signaling molecules in anticipation of future rapid activation and clonal expansion [12,13,14,15]. These metabolic patterns may indicate the presence of a population of T cells with rapid response capabilities, despite their quiescent state at the time of sampling.

In group C, there was a significant enrichment in the metabolism of arachidonic acid glycine, serine and threonine metabolism, terpenoid backbone biosynthesis, riboflavin metabolism, porphyrin and chlorophyl metabolism, steroid hormone biosynthesis, histidine metabolism, citrate cycle, glyoxylate and dicarboxylate metabolism, pentose phosphate pathway (PPP), fructose and mannose metabolism, glycolysis/gluconeogenesis, glutathione metabolism, cysteine and methionine metabolism, fatty acid elongation, purine metabolism, drug metabolism-other enzymes, metabolism of xenobiotics by cytochrome P450 (CYP), drug metabolism by CYP, one carbon pool by folate, biosynthesis of unsaturated fatty acids, beta-Alanine metabolism, N-Glycan biosynthesis, fatty acid degradation, valine, leucine and isoleucine degradation, butanoate metabolism, tryptophan metabolism, pyruvate metabolism, propanoate metabolism, pentose and glucuronate interconversions, pyrmidine metabolism and the synthesis and degradation of ketone bodies, among others. Classical metabolic pathways, including glycolysis, the PPP, the trichloroacetic acid (TCA) cycle, and oxidative phosphorylation, were active, demonstrating a high degree of metabolic plasticity. Concurrently, pathways involved in nucleotide, fatty acid, and glycosylation synthesis were also active, indicating a metabolic characteristic of simultaneous enhancement in energy production and biosynthesis. Notably, the active metabolism of glutathione and the CYP enzyme system suggested an enhancement of detoxification and oxidative defense functions. In summary, this group exhibited broad-spectrum metabolic activation, indicating that T cells are in a state of high metabolic activity.

Group D exhibited significant enrichment in metabolic pathways, including glyoxylate and dicarboxylate metabolism, PPP, fructose and mannose metabolism, glycolysis/gluconeogenesis, glutathione metabolism, cysteine and methionine metabolism, fatty acid elongation, purine metabolism, drug metabolism-other enzymes, metabolism of xenobiotics by CYP, drug metabolism by CYP, oxidative phosphorylation, glycosaminoglycan biosynthesis with heparan sulfate/heparin, vitamin B6 metabolism, folate biosynthesis, tyrosine metabolism, phenylalanine metabolism, other types of O-glycan biosynthesis, pantothenate and CoA biosynthesis, glycerolipid metabolism, glycerophospholipid metabolism, lysine degradation, selenocompound metabolism, alanine, aspartate and glutamate metabolism, arginine biosynthesis, D-Glutamine and D-glutamate metabolism, sphingolipid metabolism, inositol phosphate metabolism, phenylalanine and tyrosine and tryptophan biosynthesis (Figure 6B). Within this cluster, pathways associated with glycolysis, the PPP, and oxidative phosphorylation were notably active. Additionally, the glycerophospholipid, sphingolipid, aromatic amino acid metabolism (specifically tyrosine and phenylalanine), and glutamine/aspartate metabolism pathways were also active. This indicates a metabolic characteristic marked by the coordination of glucose and lipid metabolism alongside membrane structure remodeling. The degradation of aromatic amino acids and the metabolism of D-glutamine were notably active, potentially facilitating the supply of nitrogen sources and carbon skeletons. Furthermore, the metabolism of pantothenic acid (a precursor of coenzyme A) and vitamin B6 may play a role in maintaining redox homeostasis. This suggests that T cells may promote microenvironmental adaptive balance by remodeling the equilibrium between energy metabolism and structural synthesis. The extensive and specific metabolic profile changes observed across these different groups indicate that T cells exhibit adaptive metabolic responses in B-ALL disease, reflecting the distinct functional and activation states of T cells among the groups.

### 2.7. Comparison of T Cell and Monocyte Subsets in Four Metabolic Groups

The immunological differences among T cell and monocyte subsets across groups were analyzed using the Kruskal–Wallis test, which revealed significant immunoregulatory features in B-ALL. The results indicated that, within the monocyte subsets, group A and group C were enriched in non-classical monocytes, while group B demonstrated a stronger tendency for enrichment in the classical monocyte subset, with a higher cell proportion compared to other clusters. Group D exhibited the highest enrichment level in the intermediate monocyte subset. Notably, compared to group A, the non-classical monocytes in group D were significantly reduced (*p* < 0.05) (Figure 6D). Among T cell subsets, group A was significantly enriched in both CD8^+^ TCM and MAIT cells. Group B was specifically enriched in CD4^+^ TN cell subsets, which had a significantly higher proportion than those in other groups. Group C was specifically enriched in CD4^+^ TCM cells, with its cell proportion significantly elevated compared to other groups. Finally, group D exhibited the most extensive enrichment characteristics, being significantly enriched in CD8^+^ TN, CD8^+^ TRM, and CD4^+^ TRM cells, with all these subsets showing significantly higher proportions than those in other groups (Figure 6F).

The results underscore the intricate relationship between metabolic changes and immune alterations in B-ALL, illustrating how disruptions in metabolism can affect immune activity and possibly exacerbate disease advancement. The unique metabolic characteristics discovered among various B-ALL groups suggest that therapies targeting metabolism or modulating immune responses could be created to tackle the specific dysregulations observed in diverse B-ALL patients.

### 2.8. The Communication Between T Cells and Monocytes in B-Cell Acute Lymphoblastic Leukemia

Intercellular communication is fundamental to the regulation of the BM immune microenvironment. Our previous analysis revealed that, compared to HC cohorts, the proportion of monocytes in the BM of B-ALL cohorts exhibited significant dysregulation (Figure 3D,F). Furthermore, the composition of monocyte subsets, specifically the ratio of classical to non-classical monocytes, underwent substantial remodeling (Figure 3D,F). Given that monocytes serve as central regulators of the innate immune system, playing a pivotal role in antigen presentation, cytokine release, and T cell function regulation [5,6,7,16,17,18], we hypothesize that monocyte abnormalities may be a key factor contributing to T cell dysfunction in B-ALL.

To validate this hypothesis and explore its molecular mechanisms further, we utilized the CellChat tool to investigate the interaction network between T cells and monocytes. We focused on this specific cell interaction instead of other cell types (such as B cells, NK cells, and pDC cells) because we believe that the interaction between monocytes and T cells—mediated through pathways such as MHC, TNF, and CD40—represents the most classic and direct route of immune activation and suppression. Furthermore, this study, along with a substantial body of literature, indicates that the functional state of monocytes is closely related to the prognosis and treatment response of B-ALL patients [5,6]. Therefore, elucidating this specific interaction axis is expected to uncover the most central mechanisms of immune evasion in B-ALL.

**Figure 7:**
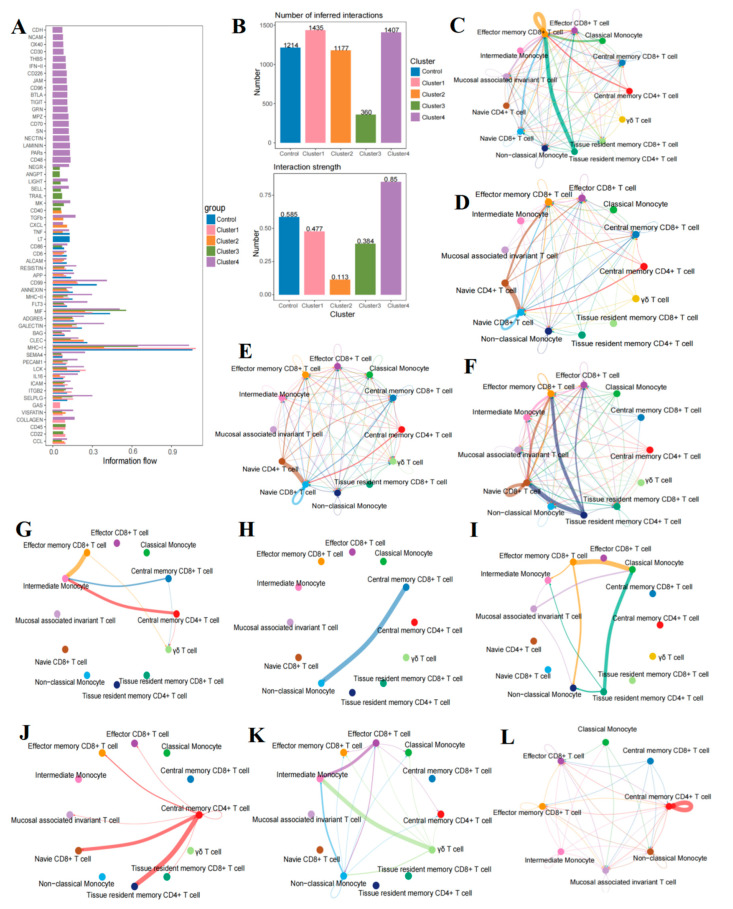
Communication between T cells and monocytes in B-ALL. (**A**) Comparison of pathway activity across B-ALL groups and healthy controls. (**B**) Number of inferred interactions by clusters. (**C**) MHC-I pathway interactions in control. (**D**) MHC-I pathway interactions in group B. (**E**) MHC-I pathway interactions in group A. (**F**) MHC-I pathway interactions in group D. (**G**) TIGIT pathway interactions in group D. (**H**) CD30 pathway interactions in group D. (**I**) TNF pathway interactions in healthy control. (**J**) CD70 pathway interactions in group D. (**K**) TNF pathway interactions in group D. (**L**) MHC-I pathway interactions in group C. Different B-ALL clusters are labeled using “Group” (e.g., Group A, Group B, etc.).

The following analysis reveals significant heterogeneity in T cell and monocyte communication across different B-ALL groups. Utilizing CellChat, we analyzed the communication differences between T cells and monocytes across four B-ALL groups and the HC cohort (Figure 7A). By quantifying the number and intensity of interactions, our results indicated that the number of interactions in the HC cohort and group A, group B, group C, and group D were 1214, 1435, 1177, 360, and 1407, respectively (Figure 7B). The interaction intensities were 0.585, 0.477, 0.113, 0.384, and 0.85, respectively (Figure 7B). These findings suggest that, compared to the HC cohort, there is significant heterogeneity in the strength and complexity of communication among different B-ALL groups. Specifically, subgroup D exhibited the highest number and intensity of interactions (number: 1407, intensity: 0.85), surpassing those in the healthy control group (number: 1214, intensity: 0.585). In contrast, subgroup C demonstrated the weakest interactions (number: 360, intensity: 0.113). This indicates that the cellular interaction landscape in B-ALL is highly complex and subtype-specific, and cannot be characterized merely as an overall enhancement or attenuation.

### 2.9. Intensive Pathway Mediation in B-Cell Acute Lymphoblastic Leukemia Group D

In group D of B-ALL, a greater number and intensity of communications were observed compared to other groups, suggesting the active involvement of multiple pathways in facilitating intercellular communication among immune cells. Notably, several signaling pathways are uniquely associated with group D, including key pathways such as CD48, CD30, and Laminin. By further concentrating on the essential signaling pathways that are closely linked to B-ALL disease progression—namely, CD30, T cell immunoglobulin and ITIM domain (TIGIT), B- and T-lymphocyte attenuator (BTLA), Laminin, CD48, type II interferon (IFN-II), thrombospondin (THBS), and CD70—we constructed their intercellular interaction networks specific to the group D (Figure 7C–L) to elucidate the distinctive intercellular interaction patterns characteristic of this B-ALL group.

Given the multifaceted roles of the tumor necrosis factor (TNF) pathway in disease progression, immune evasion, and treatment response in B-ALL, which are characterized by complex mechanisms and bidirectional regulatory properties, we have focused on the group-specific communication patterns of the TNF pathway (Figure 7I–K) [19,20]. The results indicate that, in the HC cohort, classical monocytes primarily mediate the TNF pathway through interactions with CD4^+^ TRM cells and CD8^+^ TEM cells. In contrast, within group D, this function is predominantly performed by intermediate monocytes through interactions with γδ T cells and CD8^+^ TEFF cells.

The major histocompatibility class I (MHC-I) pathway plays a crucial role in mediating communication across all groups, facilitating nearly all interactions between T cells and monocyte subsets. Among these groups, the MHC-I pathway demonstrates the highest activity in group A, followed by normal samples, while group B exhibits the lowest activity. This observation highlights the critical importance of the antigen presentation process in B-ALL, which may significantly influence patients’ autoimmune responses and overall immune function. Figure 7C–F further depict the specific cell interaction networks mediated by the MHC-I pathway in the HC cohort and each group.

Further analysis has identified additional pathways, including CD86, CD6, activated leukocyte cell adhesion molecule (ALCAM), RESISTIN, CD99, ANNEXIN, major histocompatibility complex class II (MHC-II), FMS-like tyrosine kinase 3 (FLT3), migration inhibitory factor (MIF), adhesion G protein-coupled receptor family member-E5 (ADGRE5), GALECTIN, BAG, c-type lectin-like receptor (CLEC), semaphorin 4 (SEMA4), platelet endothelial adhesion molecule 1 (PECAM1), lymphocyte-specific protein tyrosine kinase (LCK), interleukin 16 (IL16), Intercellular adhesion molecule (ICAM), selectin P ligand gene (*SELPLG*), nicotinamide phosphoribosyltransferase (VISFATIN), and c-type lectin-like molecule (CLL), etc., which mediate communication between various subsets of T cells and monocytes (Figure 7A). The pathways in question are integral to various regulatory and signaling mechanisms within the body. They significantly contribute to processes such as the control of cell adhesion and migration, inflammatory mediators and their regulatory pathways, immune response modulation, and the metabolic-inflammatory axis. The fact that these pathways are implicated across different groups of B-ALL emphasizes the intricate and dynamic nature of cellular communication in this disease. This complexity provides opportunities for targeted therapeutic interventions tailored to the unique characteristics of different B-ALL groups.

### 2.10. Analysis of Transcription Factor Activity in B-Cell Acute Lymphoblastic Leukemia Groups

**Figure 8:**
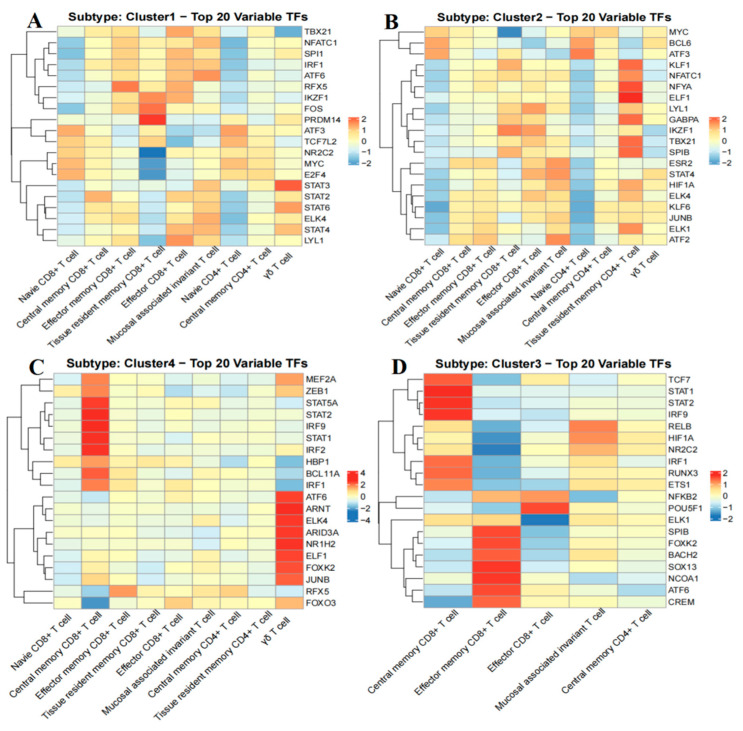
Transcription factor (TF) activation in B-ALL subtypes. (**A**) Heatmap of the top 20 TF activities with the most significant differences across cell subpopulations in group A. (**B**) Heatmap of the top 20 TF activities with the most significant differences across cell subpopulations in group B. (**C**) Heatmap of the top 20 TF activities with the most significant differences across cell subpopulations in group C. (**D**) Heatmap of the top 20 TF activities with the most significant differences across cell subpopulations in group D. Different B-ALL clusters are labeled using “Group” (e.g., Group A, Group B, etc.).

To identify the underlying molecular features driving the stratification of B-ALL metabolic groups, we analyzed the transcription factors (TFs) activities of T cell subsets across four B-ALL metabolic subtypes (Groups 1–4) based on the aggregation of all T cells, which includes the aforementioned 10 T cell subtypes. Our analysis revealed a total of 271 highly active TFs (Supplementary Table S5). The top 20 TFs exhibiting the most significant differences among cell subpopulations within each subtype were visualized using heatmaps (Figure 8A–D), indicating their potential significance in the pathogenic mechanisms underlying B-ALL.

In group A of B-ALL, the TFs nuclear factor of activated T cells c1 (NFATC1) and c-FOS protein (FOS) are closely associated with T cell activation [21], demonstrating significant activity in CD8^+^ TEM, CD8^+^ TRM, CD8^+^ TEFF, and MAIT cells. This observation indicates an active state of the adaptive immune response. Furthermore, pluripotency factor PR domain 14 (PRDM14) plays a crucial role in maintaining cellular pluripotency through epigenetic regulation [22], thereby supporting stem cell self-renewal and the undifferentiated state. Its activity in CD8^+^ TRM cells suggests that this cell subset may possess strong self-renewal capabilities, along with potential differentiation abnormalities. The elevated activity of Ikaros zinc finger (IKZF) in CD8^+^ TEM, CD8^+^ TRM, and CD8^+^ TEFF cells highlights its critical role in the regulation of lymphocyte differentiation and maturation processes. Furthermore, the pronounced activity of transcription signal transducer and activator of transcription 3 (STAT3), signal transducer and activator of transcription 4 (STAT4), and signal transducer and activator of transcription 6 (STAT6) in γδ T cells suggests their potential involvement in disease immunomodulation through the regulation of cytokine secretion and inflammatory responses. Additionally, the significant activity of TBX21 (T-bet) in CD8^+^ TCM, CD8^+^ TEM, CD8^+^ TEFF, and MAIT cells emphasizes its essential role in steering immune cells towards terminal effector differentiation and enhancing overall immune function.

In group B of B-ALL, CD8^+^ TCM, CD8^+^ TEM, CD8^+^ TRM, CD8^+^ TEFF, and CD4^+^ TRM cells exhibit similar patterns of TF activity. Among these, CD4^+^/CD8^+^ TRM cells display heightened activation of TFs, underscoring their ‘effector memory’ characteristics and their potential for rapid tumor antigen response due to long-term residence in the BM microenvironment. The elevated activity of MYC in CD4^+^/CD8^+^ TN cells suggests its crucial role in regulating the proliferation and differentiation trajectory during the early stages of T cell activation. As a core factor in T cell differentiation and immune regulation [23], the prominent activity of TBX21 across various T cell subsets, particularly in CD4^+^ TRM, indicates its significant role in driving immune surveillance, tumor cytotoxicity, and immune regulation within the BM microenvironment in this subtype.

In group C of B-ALL, the extensive activity of various TFs, including transcription factor 7 (TCF7), signal transducer and activator of transcription 1 (STAT1), signal transducer and activator of transcription 2 (STAT2), interferon regulatory factor 9 (IRF9), interferon regulatory factor 1 (IRF1), runt-related transcription factor 3 (RUNX3), ETS proto-oncogene 1 (ETS1), spi-B transcription factor (SPIB), forkhead box K2 (FOXK2), BTB domain and CNC homolog 2 (BACH2), sex-determining region Y (SRY)-box 13 (SOX13), nuclear receptor coactivator 1 (NCOA1), activating transcription factor 6 (ATF6), and cyclic AMP-responsive element modulator (CREM), in CD8^+^ TCM and CD8^+^ TEM cells indicates a potential for robust adaptive immune activation, as well as enduring tumor immune memory and regenerative capabilities within this cell population. Notably, TCF7, a core regulator of T cell memory formation and stem cell characteristics [24], exhibits significant activity in CD8^+^ TCM cells, which may directly contribute to a durable anti-tumor immune response. Furthermore, the heightened activity of POU class 5 homeobox 1 (POU5F1) in CD8+ TEFF cells suggests its critical role in sustaining long-term anti-tumor immunity by preserving residual stemness to mitigate exhaustion [25].

In the B-ALL group D, the TFs STAT1, STAT2, and IRF9 exhibit significant activity in CD8^+^ TCM cells, where they can form the interferon-stimulated gene factor 3 (ISGF3) complex in response to type I interferon signaling [26], potentially enhancing anti-tumor immune surveillance. The TF signal transducer and activator of transcription 5A (STAT5A), which is linked to the proliferation, differentiation, and functionality of immune cells [27], consistently demonstrates significant activity in CD8^+^ TCM cells, suggesting its potential role in sustaining long-term immune memory through the regulation of cell proliferation and differentiation. The elevated activity of ATF6 and aryl hydrocarbon receptor nuclear translocator (ARNT) in γδ T cells may synergistically assist these cells in adapting to the hypoxic and metabolic stresses present in the tumor microenvironment. Jun B proto-oncogene (JUNB), recognized as a core regulatory factor that limits the plasticity of γδT17 cells [28], shows significant activity in γδ T cells, potentially influencing the functions of both innate and adaptive immune responses. Furthermore, regulatory factor X5 (RFX5), a key regulator of MHC class II molecule expression [29], connects adaptive immunity through non-classical antigen presentation functions, and its heightened activity in CD8^+^ TEM cells may play a pivotal role in orchestrating their anti-tumor responses.

### 2.11. Differentially Expressed Genes Among Various Groups of B-Cell Acute Lymphoblastic Leukemia

**Figure 9:**
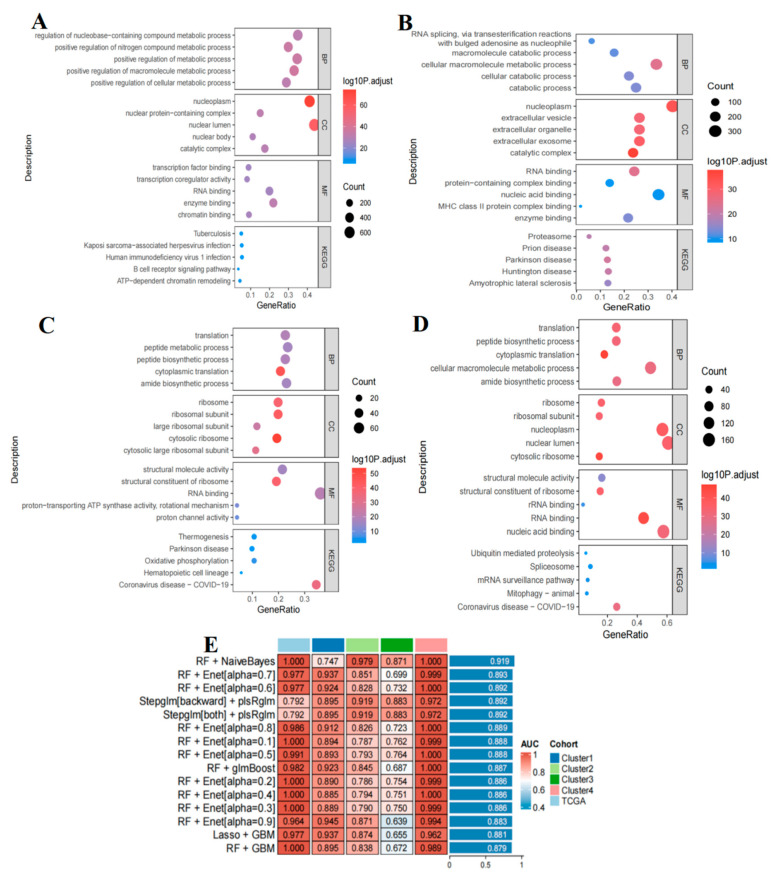
Pathway enrichment analysis in B-ALL subtypes with machine learning classification. (**A**) Bubble chart of GO and KEGG enrichment analysis in group D. (**B**) Bubble chart of GO and KEGG enrichment analysis in group C. (**C**) Bubble chart of GO and KEGG enrichment analysis in group A. (**D**) Bubble chart of GO and KEGG enrichment analysis in group B. (**E**) Performance evaluation of 113 machine learning models for precise classification of B-ALL groups based on T-Cell metabolic characteristics only displays the top 15 models (Supplementary Figure S2). Different B-ALL clusters are labeled using “Group” (e.g., Group A, Group B, etc.).

To elucidate the molecular basis of the four B-ALL metabolic groups, we compared the differentially expressed genes among the four distinct B-ALL metabolic groups within the aforementioned T-cell populations. Notably, the differential gene expression in group A is primarily associated with ribosomal function and energy metabolism, with 256 DEGs significantly enriched in pathways related to ribosome biogenesis and oxidative phosphorylation (Figure 9C, Supplementary Table S7). The enrichment of pathways involving ribosomal translation-related terms (such as translation, ribosome, cytosolic ribosome, and peptide biosynthetic processes) and structural constituents of ribosomes indicates abnormally elevated protein synthesis in this subtype, driving the malignant proliferation of leukemia cells. Additionally, the dysregulation of ribosome biogenesis is often closely linked to the development and progression of diseases such as cancer [30,31]. The significant enrichment of oxidative phosphorylation and thermogenesis further corroborates its reliance on high-energy metabolism to sustain the malignant phenotype. Numerous studies have established that oxidative phosphorylation is crucial to the pathogenicity and drug resistance of B-ALL cells [32]. This process not only provides essential energy for the proliferation and survival of leukemia cells but also, when inhibited, can restore cellular sensitivity to chemotherapeutic agents, thereby enhancing treatment efficacy. Consequently, the development of new therapeutic targets aimed at the oxidative phosphorylation pathway is theoretically justified, and future interventions targeting this pathway are anticipated to improve clinical outcomes for this subtype. Additionally, the enrichment of the hematopoietic cell lineage pathway indicates that this subtype may be associated with a blockade in early B-cell differentiation. Hematopoietic lineage abnormalities play a critical role in the pathogenesis and treatment of B-ALL [33]. B-ALL arises from B-lineage lymphoid progenitor cells, which proliferate abnormally within the BM and inhibit normal hematopoiesis, resulting in anemia, thrombocytopenia, and neutropenia [34]. Common genetic alterations, such as the breakpoint cluster region-Abelson (*BCR-ABL1*) fusion gene [35], can lead to the aberrant differentiation of hematopoietic lineages, causing excessive proliferation of B-lineage cells and a loss of their normal differentiation capacity. Comprehensive research into the mechanisms and clinical significance of these lineage abnormalities will yield important insights for the precise diagnosis, treatment, and prognostic evaluation of B-ALL.

The differential gene expression in group B is primarily linked to RNA processing and protein degradation, characterized by 296 DEGs that display abnormalities in RNA processing and imbalances in protein homeostasis (Figure 9D, Supplementary Table S7). The enrichment of the spliceosome and mRNA surveillance pathways indicates the presence of spliceosome mutations or defects in RNA processing. Mutations in RNA splicing regulatory factors are commonly observed across various cancers and can lead to recurrent aberrant splicing of mRNA isoforms [36]. For example, splicing factor genes such as serine/arginine-rich splicing factor 2 (*SRSF2*) and zinc finger CCCH-type, RNA-binding motif and serine/arginine-rich 2 (*ZRSR2*) are frequently mutated in myeloid neoplasms [37], triggering specific splicing abnormalities that result in aberrant mRNA isoforms translated into tumor-specific neoantigens. Given that such mutations are shared among similar patients, they may serve as broad-spectrum therapeutic targets. Meanwhile, the enrichment of the ubiquitin-mediated proteolysis pathway indicates dysregulation of proteasomal degradation. Aberrant ubiquitination of certain key proteins in B-ALL cells, such as mutations in the paired box protein 5 (*PAX5*) gene that may affect its ubiquitination modification and interfere with B-cell differentiation [38], can lead to erroneous degradation or accumulation of these proteins. Furthermore, the enrichment of pathways related to nucleoplasm localization, nuclear lumen, nucleic acid binding, rRNA binding, and translation supports the existence of intranuclear RNA metabolism disorders in this group. This disorder, alongside proteasome degradation dysregulation, results in the accumulation of abnormal proteins, thereby activating cell proliferation signals that drive malignant transformation. The characteristics of these enriched signaling pathways confirm that proteasome degradation dysregulation is a key mechanism in the onset and progression of B-ALL group B. Consequently, therapeutic strategies targeting the proteasome offer a promising direction for the precision treatment of this subtype.

The differential gene expression in group C is primarily associated with the proteasome and extracellular vesicles, including 900 DEGs that are predominantly involved in proteasome-mediated protein degradation and extracellular vesicle secretion (Figure 9B, Supplementary Table S7). The enrichment of pathways such as RNA splicing, proteasome function, and macromolecule catabolic processes indicates a significant imbalance in protein homeostasis, which may lead to the accumulation of misfolded proteins and the formation of aggregates within B-ALL cells. This accumulation can interfere with normal cellular functions; for instance, the misfolding of specific leukemia-associated proteins may result in the loss of their normal functions or the acquisition of abnormal activities, thereby promoting the proliferation, survival, invasion, and metastasis of leukemia cells [39,40]. Furthermore, the notable enrichment of the extracellular vesicle/exosome signaling pathway suggests active intercellular communication within this group. Extracellular vesicles serve as crucial mediators of intercellular communication and may play significant roles in immune evasion, angiogenesis, and microenvironment modulation during tumorigenesis and tumor development [41,42]. This suggests that tumor cells utilize exosomes to facilitate disease progression. Furthermore, the enrichment of signaling pathways related to MHC-II complex binding and enzyme activity indicates a potential involvement of abnormal immune responses in disease progression [43]. Under normal conditions, the expression and function of MHC-II complexes are vital for the immune system’s ability to recognize and eliminate pathogens and abnormal cells. Impairment of their function may result in inadequate antigen presentation, hindering the immune system’s capacity to effectively eliminate tumor cells [44]. Therefore, conducting in-depth research on the role of MHC-II complexes in this group could enhance our understanding of the pathogenesis of B-ALL and provide a theoretical foundation for the development of novel therapies.

The differential gene expression in group D is primarily associated with B cell receptor (BCR) signaling and epigenetic regulation, encompassing 1656 DEGs predominantly influenced by BCR signaling and epigenetic remodeling (Figure 9A, Supplementary Table S7). In B-ALL cells, the BCR signaling pathway is often aberrantly activated due to genetic mutations or chromosomal translocations, resulting in uncontrolled cell proliferation and resistance to apoptosis [45]. This enrichment directly reflects the abnormal activation of malignant B cells. Moreover, the enrichment of ATP-dependent chromatin remodeling and TF binding pathways suggests the presence of epigenetic silencing, which may inhibit tumor antigen presentation, promote immune escape, and facilitate the growth and survival of B-ALL cells by affecting intercellular signaling pathways [46,47]. Furthermore, studies indicate that epigenetic remodeling can regulate glycolysis-related genes, such as the ubiquitin-like with PHD and ring finger domains 1 (UHRF1) protein, which modulates the expression of glycolytic genes through epigenetic mechanisms, thereby promoting aerobic glycolysis in B-ALL cells and providing energy support for cell proliferation [48]. Finally, the enrichment of pathways related to infectious diseases, including tuberculosis, HIV, and Kaposi’s sarcoma-associated herpesvirus, suggests that patients with this subtype may have acquired immune deficiency, which increases the risk of opportunistic infections.

### 2.12. Advanced Machine Learning Models for Subtype Classification

Subsequently, we developed a machine learning classification model based on T-cell metabolic characteristics to achieve precise differentiation of B-ALL groups. The input features for the model were derived from KEGG metabolic pathway scores at the single-cell level (Supplementary Table S6). We evaluated a total of 113 model combinations, which demonstrated robust predictive capabilities, achieving AUC values ranging from 0.792 to 1 in the training set (Figure 9E) (Supplementary Table S6 and Figure S2). Notably, this high performance extends to the validation set, particularly for high-performance models such as RF+NaiveBayes, RF+Enet[alpha = 0.7], RF+Enet[alpha = 0.6], Stepglm [backward]+plsRglm, and Stepglm [both]+plsRglm (Figure 9E, Supplementary Figure S2). Among these, the RF+NaiveBayes model stands out with the highest average AUC value, and its lowest AUC across multiple dataset validations remains at 0.747 (Figure 9E, Supplementary Figure S2). Its overall performance significantly surpasses that of other combinations and likely represents an optimal balance between stability and accuracy. The high predictive accuracy of the model underscores the application value of advanced computational technologies in the precise subtyping of B-ALL, providing a reliable computational tool for determining precise subtypes of B-ALL based on T-cell metabolic profiles, and is expected to assist in formulating individualized treatment plans in clinical practice.

### 2.13. Drug Enrichment Analysis for Personalized Treatment of B-Cell Acute Lymphoblastic Leukemia Subtypes

**Figure 10:**
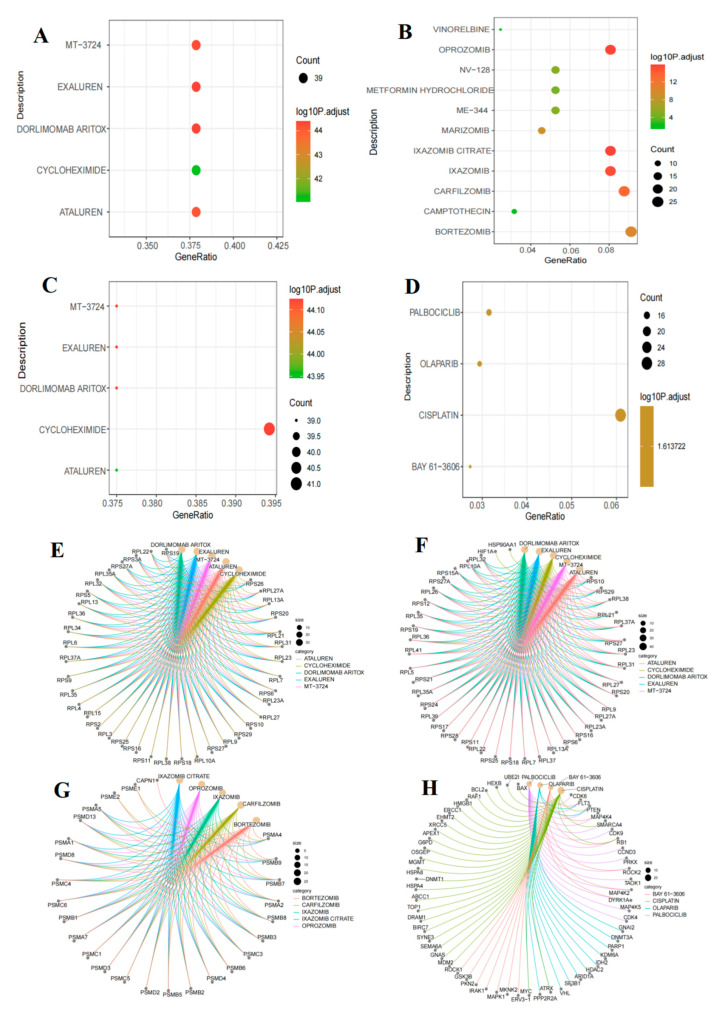
Drug enrichment analyses for B-ALL subtypes. (**A**) The most significant enriched drug results in group A. (**B**) The most significant enriched drug results in group B. (**C**) The most significant enriched drug results in group C. (**D**) The most significant enriched drug results in group D. (**E**) Genes associated with drug enrichment in group A. (**F**) Genes associated with drug enrichment in group B. (**G**) Genes associated with drug enrichment in group C. (**H**) Genes associated with drug enrichment in the group D. Different B-ALL clusters are labeled using “Group” (e.g., Group A, Group B, etc.).

To advance the application of the four B-ALL groups in the development of precision treatment strategies, we conducted a drug enrichment analysis on the DEGs within each group (Figure 10A–D, Supplementary Table S8). This analysis aimed to identify potential therapeutic drugs tailored to the specific needs of each B-ALL group, thereby providing a foundation for formulating precision treatment strategies. Furthermore, we constructed drug-associated gene interaction networks for each group, which visually illustrate the key molecular targets of drug actions, thereby offering a molecular basis for elucidating the mechanisms of drug action (Figure 10E–H, Supplementary Table S8).

In group A of B-ALL, Dorlimomab Aritox has been identified as the most significantly associated therapeutic agent (Figure 10A). The drug–gene interaction network analysis revealed that its key targets are significantly enriched in ribosomal protein genes, such as ribosomal protein L22 (*RPL22*), ribosomal protein S19 (*RPS19*), and ribosomal protein S34 (*RPS34*) (Figure 10E). This finding is consistent with the characteristics of aberrant ribosome biogenesis in group A. As the critical site for protein synthesis, ribosomal dysfunction may lead to disordered protein synthesis in leukemia cells. The overactivation of ribosomal genes promotes malignant progression by providing essential ribosomal support for abnormal proliferation [49,50]. Furthermore, the strong association of Dorlimomab Aritox with these genes underscores its therapeutic potential.

In group B, Dorlimomab Aritox was reaffirmed as a critical drug (Figure 10B), with its mechanism of action potentially involving the direct inhibition of malignant proliferation and disease progression in leukemia cells through targeting ribosomal proteins (Figure 10F). In group C, IXAZOMIB CITRATE, a proteasome inhibitor, emerged as the most significantly associated drug (Figure 10C), with its targets notably enriched in immunoproteasome subunits, such as proteasome subunit-beta type-7 (PSMB7) and proteasome subunit-beta type-9 (PSMB9) (Figure 10G). This finding aligns closely with the pathological characteristics of proteasome degradation dysregulation observed in group C, underscoring its therapeutic translational value.

For group D, palbociclib has been identified as one of the most promising therapeutic agents for B-ALL (Figure 10D). As the first cyclin-dependent kinase 4/6 (CDK4/6) inhibitor approved by the FDA, it directly targets CDK4/6 by blocking the CDK4/6-cyclin D complex and inhibiting Rb protein phosphorylation, which leads to cell cycle arrest at the G1 phase. Studies have found that CDK4 and CDK6 are frequently overexpressed in pediatric B-ALL patients, with cancer cells relying on this pathway for excessive proliferation [51]. Targeted inhibition can induce significant apoptosis, demonstrating substantial therapeutic potential. Preclinical studies, such as those using ribociclib in combination with dexamethasone, have shown that CDK4/6 inhibitors, including palbociclib, effectively inhibit the proliferation of B-ALL cell lines (including dexamethasone-resistant cell lines SEM and RCH-ACV) and patient-derived primary cells, inducing G1 phase arrest [51]. The clinical efficacy of this treatment strategy has also been preliminarily validated: palbociclib combined with dexamethasone has exhibited targeted effects in patients with relapsed or refractory B-ALL, such as reduced expression of CD34^+^ cells, p-RB, c-MYB, and BCL-2 [52]. In another study, palbociclib combined with reinduction chemotherapy was well tolerated in patients with relapsed/refractory B/T-cell ALL and lymphoma, with complete remission observed in heavily pretreated patients [53]. Furthermore, palbociclib is closely associated with key genes of group D (such as cyclin D3 (*CCND3*), *CDK4*, *CDK6*, FMS-like tyrosine kinase 3 (*FLT3*), mitogen-activated protein 4 kinase 4 (*MAP4K4*), etc.) (Figure 10H) [54], further indicating its broad application prospects in the future treatment of this group.

## 3. Discussion

B-ALL is a malignant neoplastic disease that originates from B-lineage lymphoid precursor cells, characterized by the rapid proliferation of tumor cells and resistance to treatment [1]. This study aims to uncover the functional abnormalities of the immune system and significant changes in metabolic pathways associated with the disease by analyzing scRNA-seq data from BM tissue samples of B-ALL patients and healthy individuals. The results indicate notable alterations in immune cells within the BM of B-ALL cohorts, including a significant increase in Pro-B cells and a marked decrease in B cells, NK cells, monocytes, and pDCs. In B-ALL cohorts, a significantly lower proportion of monocytes is observed compared to the normal range. This reduction may be related to the suppression of normal hematopoiesis, particularly the generation of monocyte lineages, due to the excessive proliferation of leukemia cells. Furthermore, the abnormalities of monocytes in B-ALL not only reflect the disease status but also contribute to disease progression and influence treatment prognosis [5,6]. Research has demonstrated that B-ALL cells modify vascular structures by remodeling the BM microenvironment, which in turn promotes monocyte differentiation [5]. Targeting the elimination of leukemia-associated monocytes has been shown to prolong remission periods. Non-classical monocytes (CD14^+^CD16^++^) that exhibit high expression levels of CX3C motif chemokine receptor 1 (CX3CR1) are significantly elevated in the peripheral blood of B-ALL patients, and their abnormal recruitment is directly associated with BM stromal remodeling, which is mediated by the upregulation of CX3C chemokine ligand 1 (CX3CL1) in the BM [6]. Furthermore, normal monocyte counts at the time of relapse correlate with improved survival rates in adult patients with early relapsed B-ALL [55]. Additionally, a significant increase in the proportion of Pro-B cells may directly indicate the malignant clonal expansion of B-ALL [56]. The significant reduction in the proportion of mature B cells suggests that the malignant proliferation of B-ALL cells, along with the suppression of the immune microenvironment, hampers the differentiation and maturation of normal B cells [57]. Furthermore, the marked decrease in the proportions of NK cells, monocytes, and pDCs provides further evidence that the innate immune surveillance function in B-ALL patients is severely compromised. This impairment may contribute to disease progression, including diminished tumor clearance capacity, delayed immune responses, and an increased risk of recurrence [58,59,60].

Based on the annotation of marker genes for T cell subsets, we identified a total of ten distinct T cell subsets: CD8^+^ TN, CD8^+^ TCM, CD8^+^ TEM, CD8^+^ TRM, CD8^+^ TEFF, MAIT, CD4^+^ TN, CD4^+^ TCM, CD4^+^ TRM, and γδ T cells. Further evaluation of the differences in the proportions of various T cell subsets between B-ALL cohorts and HC cohorts revealed that the proportions of CD8^+^ TEM, CD8^+^ TEFF, and MAIT cells were significantly lower in B-ALL cohorts compared to the HC cohorts. This finding suggests a diminished capacity of the immune system in these patients to monitor and eliminate B-ALL cells, thereby highlighting the close association between severely impaired anti-tumor immune function and immune evasion in B-ALL [61,62]. CD8^+^ TEM cells persist within tumor tissues and fulfill long-term immune surveillance roles, enabling their rapid activation to eliminate recurrent tumors [63]. A reduction in these cells directly compromises tumor clearance capabilities. Consequently, the decrease in CD8^+^ TEM cells observed in B-ALL cohorts may indicate a long-term functional impairment of the immune system, thereby increasing the risk of tumor recurrence or infections. CD8^+^ TEFF cells induce tumor apoptosis through the perforin-granzyme/Fas pathway; thus, a decline in their proportion leads to the exhaustion of cytotoxic responses, which directly impacts tumor development and treatment efficacy [64]. MAIT cells act as a bridge between innate and adaptive immunity, allowing for rapid responses to microbial and tumor antigens. Their absence diminishes local immune surveillance in the mucosa and BM, potentially facilitating immune evasion by leukemia cells [65]. Furthermore, the tumor microenvironment, through persistent antigen stimulation, drives the differentiation of CD8^+^ TEM cells into exhausted T cells characterized by high expression of PD-1/TIM-3, which is accompanied by a loss of effector functions [66,67]. Furthermore, we observed that certain annotated cell subsets formed multiple discrete clusters on the UMAP plot, such as CD4^+^ TCM cells depicted in Figure 3A and intermediate monocytes shown in Figure 3D. This finding indicates that, within the pathological context of B-ALL, traditionally defined immune cell subsets may comprise functionally distinct subpopulations. These subpopulations could arise from different clones or exist in varying states of functional polarization, collectively contributing to the complexity of the tumor immune microenvironment. The imbalance in the proportions of these T cell subsets may stem from the combined effects of the tumor microenvironment, immune suppression, cytokine imbalance, and metabolic disorders, ultimately exacerbating the progression of B-ALL [11].

Our pseudotime analysis revealed a continuous spectrum of functional state changes in T cells within the bone marrow microenvironment of B-ALL. It is crucial to emphasize that the arrangement of CD4^+^ and CD8^+^ T cell states in the trajectory map is based on their transcriptomic similarity and does not imply transdifferentiation between lineages. CD4^+^ and CD8^+^ TN cells cluster at the starting point of the trajectory due to their shared quiescent state characteristics, subsequently entering distinct functional state differentiation branches: CD8^+^ T cells tend to differentiate into terminal effector subsets with cytotoxic and tissue-resident properties, while CD4^+^ T cells exhibit independent pathways leading to the generation of TCM cells or TRM cells. Future studies, such as those utilizing lineage tracing techniques, will help verify whether these distinct subsets indeed converge to the same functional endpoint. This discovery underscores the extensive reprogramming of T cell functional states by the B-ALL microenvironment, which may collectively contribute to the failure of anti-tumor immune functions.

Furthermore, our pseudotime analysis delineates a distinct trajectory of T cell state transitions within the B-ALL tumor microenvironment. In contrast to the canonical T cell differentiation patterns observed in healthy organisms [68,69], the trajectory revealed in this study exhibits several notable aberrant features. Firstly, in a healthy context, CD4^+^ and CD8^+^ TN cells typically differentiate into relatively distinct effector and memory cell subsets following antigen stimulation. However, in the context of B-ALL, we observed that the effector/cytotoxic subsets (such as TEX and TRM) and gene modules associated with exhaustion or dysfunctional states (such as SOCS3) exhibited earlier co-expression or overlap along the developmental trajectory. This suggests that the tumor microenvironment may induce T cell exhaustion prematurely, thereby interfering with their normal effector differentiation program. Secondly, the unique differentiation pathway of CD4^+^ TCM, which is independent of the main effector branch, may reflect the distinctive reprogramming effects of specific signals in the microenvironment (such as persistent antigen stimulation or inhibitory cytokines) on the fate determination of CD4^+^ T cells. Collectively, these deviations from the classical model of healthy differentiation characterize the immunosuppressive microenvironment of B-ALL and may be a key factor leading to the failure of anti-tumor immune functions [70]. State 2 predominantly differentiates into effector and tissue-resident subsets, including CD8^+^ TRM, CD8^+^ TEFF, MAIT, CD4^+^ TRM, and γδ T cells. This suggests a shared differentiation pathway for effector functions and tissue-residency characteristics, potentially governed by common regulatory mechanisms. In contrast, State 3 differentiates exclusively into CD4^+^ TCM cells, indicating its independence from the effector branch and possible regulation by distinct signals. The effector and cytotoxic subsets, such as CD8^+^ TEFF cells, are concentrated at the terminal end of the State 2 branch, which aligns with their terminally differentiated status [71]. Moreover, the tissue-resident subsets (CD4^+^/CD8^+^ TRM) share the same branch as effector cells, reinforcing the notion that TRM cells originate from effector precursors [72,73]. Gene module GO enrichment and key gene clustering analyses revealed that genes exhibiting variation with developmental time can be categorized into five clusters. These clusters are involved in protein synthesis (ribosomal function), oxygen transport and metabolism, immune cell effector functions (including immune killing and cytotoxicity), ATP synthesis and proton transport (mitochondrial energy metabolism), as well as the core functions of ATP synthase. The relative expression of key genes along the pseudotime trajectory indicated that in cell fate 1 (the effector branch), the expression of *GZMH* and *NKG7* genes was elevated, reflecting an upregulation of cytotoxicity. Conversely, the expression of *ARHGAP45* and *SELL* genes was reduced, indicating decreased homing and migration capabilities, which aligns with the tissue-resident characteristics of effector T cells [74,75]. In cell fate 2 (the memory branch), the expression of *SOCS3* and *LEF1* genes was upregulated, promoting the maintenance of homeostasis [76,77], while the expression of the *ARHGAP45* gene was downregulated [74]. This alteration may facilitate long-term cell survival and enhance circulating memory functions. In summary, these findings provide potential targets for the precise regulation of T cell fate, such as enhancing anti-tumor effects and optimizing vaccine memory responses in B-ALL.

To further evaluate the impact of different proportions of T cell subsets on the prognosis of patients with B-ALL, KM curve analysis demonstrated significant associations between CD8^+^ TN, CD8^+^ TEFF, CD4^+^ TRM, and γδ T cells with the prognosis of B-ALL patients. Given the critical roles of these T cell subsets in tumor response [78,79], future studies are warranted to comprehensively understand their impact on B-ALL treatment, particularly in the context of immunotherapy.

Multiple studies have confirmed that the metabolic reprogramming of T cells within the tumor microenvironment is a fundamental mechanism of immune evasion. This process involves multi-level dysregulation, including glycolysis, amino acid metabolism, and oxidative phosphorylation [80]. The tumor microenvironment induces a metabolic imbalance in T cells, resulting in a sustained loss of function [81]. For instance, the suppression of mitochondrial function in CD8^+^ T cells is characterized by impaired oxidative phosphorylation and reduced mitochondrial quality [82]. Research on the metabolic heterogeneity of T cell subsets has elucidated the specific metabolic profiles of different B-ALL patients. In group A, T cells demonstrate active oxidative phosphorylation and catabolism of fatty acids and amino acids, indicating a reliance on catabolic processes. This may reflect an adaptive reprogramming towards catabolism in nutrient-deprived environments, which could be accompanied by functional exhaustion, thereby limiting anti-tumor immune responses [83]. Group B is characterized by active fatty acid synthesis and the biosynthesis of sphingolipids, steroids, and O-glycans. Notably, this anabolic pattern aligns closely with the fatty acid oxidation-dependent properties of T regulatory (Treg) cells [84], which may further promote an immunosuppressive microenvironment. Group C exhibits extensive metabolic reprogramming, evidenced by simultaneous enhancements in glycolysis, the PPP, the TCA cycle, oxidative phosphorylation, nucleotide and fatty acid synthesis, glycosylation, glutathione metabolism, and P450 systems. This suggests that T cells are in a highly active state with significant metabolic plasticity, driving immune activation [85]. Group D displays metabolic features indicative of an adaptive balance in T cell metabolism, potentially achieved through the reconstruction of multifunctional metabolic modules that enable adaptability to the tumor microenvironment. We discovered that a subset of T cells exhibiting active anabolic metabolism (designated as group B) did not enter the cell cycle functionally. This observation reveals a phenomenon of ‘decoupling’ between T cell metabolism and proliferation within the tumor microenvironment. One potential explanation for this is that the tumor microenvironment provides inhibitory signals, such as PD-1 and CTLA-4, which prevent these cells from progressing through the division cycle despite their metabolic capabilities, resulting in a state of ‘functional entrapment.’ Alternatively, these metabolic alterations may represent an adaptive response of T cells to microenvironmental stresses, including hypoxia and nutrient deprivation [83,86]. This finding underscores the importance of integrating the functional states of cells when analyzing metabolic data for a comprehensive interpretation. Therefore, understanding these metabolic alterations will provide a foundational metabolic profile for the stratified treatment of B-ALL patients and offer novel strategies for developing targeted therapies aimed at remodeling the anti-tumor functions of T cells.

Abnormalities in intercellular communication pathways were observed in B-ALL cohorts, which may be linked to heightened immune responses [87]. We have conducted a thorough investigation into the highly dysregulated interaction axis between monocytes and T cells in B-ALL. While the proportions of other immune cells, such as NK cells and pDC cells, also exhibit changes and undoubtedly contribute to microenvironmental regulation, our data emphasize that the reprogramming of monocytes and their aberrant communication with T cells represent a distinctive component of the immunosuppressive microenvironment in B-ALL. Future research focused on constructing a comprehensive interaction network that encompasses all myeloid and lymphoid cells will provide a more holistic perspective. Our analysis of cell communication indicates that cellular interactions within the BM microenvironment of B-ALL are not uniformly enhanced; rather, they display a significant degree of heterogeneity and specificity based on distinct groups. Cellchat analysis indicated increased activity in pathways such as TIGIT, CD30, and CD70 in group D. These molecular networks play a crucial role in the body’s immune regulatory mechanisms, particularly in promoting T cell activation and mediating cytokine signal transduction processes, which have significant biological implications [67,88,89]. For example, CD30 is highly expressed in various lymphomas, including Hodgkin’s lymphoma and certain subsets of diffuse large B-cell lymphoma. Its chronic signaling promotes the expansion of B1 cells and plasma cells in B-cell lymphomas, facilitating differentiation through STAT3/6 phosphorylation and contributing to lymphomagenesis, thereby suggesting its involvement in aggressive B-cell malignancies [88]. Interferon-gamma (IFN-γ) is secreted by activated T cells and NK cells, playing a crucial role in tumor progression through bidirectional regulation of immune responses. While it exerts antitumor effects, it also facilitates tumor progression by inducing immune escape mechanisms such as PD-L1 expression [90,91]. TIGIT, an immune checkpoint receptor, is co-expressed with PD-1 and LAG3 in exhausted T cells of patients with B-ALL [67], synergistically inhibiting T cell function. This finding suggests that the combined blockade of TIGIT and PD-1 may reverse immune exhaustion and enhance antitumor responses [92]. CD70 is markedly expressed in hematological malignancies, activating signaling pathways via its interaction with CD27, which mediates T cell exhaustion, enhances Treg function, and recruits tumor-associated macrophages. The blockade of this axis can improve the infiltration of T/NK cells and enhance therapeutic efficacy [89]. The TNF signaling pathway significantly influences the progression of B-ALL by regulating cell survival, inflammatory responses, and immune cell activation [93,94]. In contrast, the MHC-I pathway, a fundamental component of immune surveillance and evasion, underscores the critical role of antigen presentation and recognition in B-ALL, as evidenced by its distinct expression patterns across four groups [95]. This highlights new opportunities for targeted therapy. In summary, these findings collectively elucidate the complexity of the immune regulatory network in B-ALL and the potential for targeted interventions.

Focusing on the significant role of TFs in the function and metabolic processes of immune cells in B-ALL, it has been observed that multiple TFs exhibit differential expression across various B-ALL subtypes. Those involved in immune cell differentiation, functional regulation, and metabolic adaptation are particularly prominent. Notably, *EBF1*, a key regulator of CD22 expression during B-cell differentiation, shows aberrant expression or dysfunction in B-ALL, leading to abnormal CD22 expression and affecting sensitivity to CD22-targeted therapies [96]. Additionally, myocyte enhancer factor-2D (MEF2D) rearrangements, such as the MEF2D-HNRNPU1 (*MH*) fusion, aberrantly activate MEF2D’s transcriptional activity, disrupt the expression of genes related to B-cell development, and promote the malignant proliferation of leukemia cells [97]. In the case of MEF2D-rearranged B-ALL, targeting its downstream regulatory factors, such as histone deacetylase 9 (HDAC9), may represent a more effective therapeutic strategy [98]. These findings open new avenues for addressing the dysregulation of TF activity in B-ALL. By focusing on specific TFs, it is feasible to restore immune homeostasis and enhance metabolic regulation, thereby providing more precise solutions for personalized treatment.

The drug enrichment analysis results indicate that the integration of TF regulatory networks with alterations in metabolic pathways for formulating individualized therapies for B-ALL presents significant application prospects. This analysis reveals that Dorlimomab Aritox is closely associated with the expression of specific ribosomal protein genes and biosynthesis pathways, suggesting its potential therapeutic value by targeting dysfunctional protein synthesis and abnormal ribosomal function in B-ALL. Additionally, the inhibitor palbociclib, which targets the CDK4/6-cyclin D complex pathway, demonstrates substantial therapeutic potential for B-ALL, with its efficacy validated in multiple preclinical and clinical studies [53,54]. These findings emphasize the importance of integrating TF activity profiles with drug target information to optimize individualized treatment regimens. Therefore, precision intervention strategies based on patient-specific molecular pathological features hold promise for enhancing the clinical efficacy of B-ALL.

Machine learning techniques that leverage T-cell metabolic characteristics offer a novel diagnostic tool for the classification of B-ALL subtypes, with high AUC values affirming the model’s reliability in clinical practice. This groundbreaking advancement not only facilitates precise patient stratification but also provides scientific evidence for the development of personalized treatment plans. By conducting an in-depth analysis of B-ALL subtype-specific metabolic markers and immune features, medical teams can devise more targeted therapeutic strategies tailored to the specific pathological mechanisms of patients, thereby significantly enhancing the effectiveness of clinical interventions.

However, this study has several limitations. First, the analysis is entirely based on publicly available datasets, and the limited sources may result in insufficient data representativeness and generalizability. Second, the machine learning model was constructed and validated only on a limited sample, and it has not yet been validated in large-scale independent cohorts (such as the TARGET database) or prospective clinical trials, indicating that its clinical applicability requires further evaluation. The machine learning classifier developed in this study is based on single-cell metabolic profiles. Although it has demonstrated excellent performance in internal datasets, it has yet to be validated in independent external cohorts. This limitation arises primarily because the metabolic pathway activity features of pure T-cell populations cannot be extracted from conventional bulk RNA-seq data, rendering the direct application of this model unfeasible. Future research must focus on acquiring new B-ALL single-cell RNA-seq datasets to further validate the reliability and clinical prognostic value of this metabolic classification system, thereby facilitating its translation into clinical applications. Additionally, intratumoral heterogeneity and individual patient differences may also affect the accuracy and consistency of the results. More importantly, the computationally predicted candidate therapeutic targets (such as the ribosome/proteasome pathway) and potentially effective drugs (such as Dorlimomab Aritox and IXAZOMIB CITRATE) have not been experimentally validated. In the future, the anti-leukemia activity and mechanisms of action of these targets and drugs need to be rigorously tested through in vitro cell experiments, gene function interference studies, and in vivo patient-derived xenograft (PDX) model experiments.

It is noteworthy that the publicly available databases we utilized did not provide detailed clinical baseline information about the patients, such as age, sex, and precise genetic subtypes of B-ALL (e.g., Ph+, Ph-like, MLL rearrangements). Consequently, we were unable to assess whether these crucial baseline characteristics were balanced between the B-ALL group and the healthy control group, nor could we entirely rule out their potential impact on the observed variations in immune cell proportions, metabolic heterogeneity, or patient prognosis. Future studies with a prospective design are necessary to include cohorts with complete clinical annotations to validate whether our proposed metabolic classification system is independent of these traditional clinical factors, thereby further confirming its independent value for clinical application. Despite the aforementioned limitations, this study provides a valuable clinical decision-making basis for the treatment strategies of B-ALL patients and lays an important foundation for subsequent research. Only through more extensive experimental validation and prospective clinical studies can the classification strategies and treatment directions proposed in this study ultimately be applied to clinical precision treatment practices.

## 4. Materials and Methods

### 4.1. Data Collection

scRNA-seq data from BM tissues of patients with B-ALL and healthy individuals were sourced from the GEO database (https://www.ncbi.nlm.nih.gov/geo/, accessed on 4 August 2025) [99], including GSE130116, GSE132509, GSE185381, and GSE200557, comprising a total of 17 B-ALL patients (B-ALL cohorts) and 13 healthy control (HC) cohorts. Transcriptomic and survival data for B-ALL patients were downloaded from the UCSC GDC Xena Hub (https://gdc.xenahubs.net) [100], which contains 135 B-ALL samples, while the survival dataset includes 86 B-ALL samples. After integration, 47 matched B-ALL samples with both transcriptomic and survival data were identified.

### 4.2. Single-Cell RNA Sequencing Quality Control and Cell-Type Annotation

All single-cell data were analyzed using the R package Seurat (Version 4.4.0, https://satijalab.org/seurat/, accessed on 1 August 2025) [101]. Low-quality cells, defined as those containing fewer than 200 genes or having fewer than 5 cells expressing a given gene, were filtered out. Additionally, cells with fewer than 500 or more than 3500 expressed genes, as well as those with a mitochondrial gene proportion exceeding 5%, were excluded. The single-cell data were normalized using the NormalizeData function, and highly variable genes exhibiting significant variation across cells were identified using the FindVariableFeatures function. The top 2000 highly variable genes, which demonstrated substantial fluctuations, were selected for subsequent analysis. Following this, the single-cell data were scaled using the ScaleData function, and principal component analysis (PCA) was performed based on the top 2000 highly variable genes. The RunHarmony function was subsequently applied to integrate multiple scRNA-seq datasets. Clustering analysis was conducted using the FindClusters and FindNeighbors functions from the Seurat package, with a resolution parameter set to 0.3, and two-dimensional visualization was achieved using Uniform Manifold Approximation and Projection (UMAP).

In the initial clustering and cell type annotation analysis of this study, we intentionally included T cell receptor (TCR), immunoglobulin (IG), and human leukocyte antigen (HLA) genes. This decision is based on the critical role that TCR and IG genes play as markers for defining T lymphocyte and B lymphocyte lineages; their specific expression forms the fundamental basis for distinguishing these major cell types. Excluding these genes would jeopardize the accuracy of cell type identification and could hinder our ability to effectively differentiate important immune cell populations. To ensure that the clustering results accurately reflect broad biological heterogeneity rather than being dominated by a limited number of genes, our clustering analysis is strictly based on the top 2000 highly variable genes (HVGs). This selection process naturally diminishes the influence of genes that do not exhibit high variability in expression across cells, including those that are consistently highly expressed within specific cell populations. Subsequently, sub-clustering analyses for specific cell populations (e.g., T cells, B cells) are performed within more homogeneous subsets of cells, facilitating a more refined resolution of cell states without excessive influence from lineage marker genes.

To identify cell types in the B-ALL and HC cohorts from the single-cell dataset, we first employed the FindAllMarkers function (Seurat package) within each dataset to pinpoint the highly expressed genes (marker genes) of each cell cluster in comparison to all other clusters (thresholds: logfc.threshold = 0.25, min.pct = 0.25, only.pos = TRUE). This analysis seeks to uncover transcriptomic features that define the identity of each cluster while ensuring that comparisons are made between transcriptionally similar clusters. Subsequently, we utilized the R package SingleR (Version = 2.2.0, https://www.bioconductor.org/packages/release/bioc/html/SingleR.html, accessed on 2 August 2025) [102] to annotate the cell types of the clusters and integrated the results of these cluster-specific marker genes with widely recognized classic cell marker genes from existing literature [5,103]. Based on these criteria, we classified the 20 computational clusters into 8 major biological cell types.

### 4.3. T Cell and Monocyte Clustering and Annotation

To identify the cellular subpopulations within T cell subsets, the previously described steps—normalization, dimensionality reduction, and clustering—were applied to T cell types, resulting in the identification of 16 distinct T cell subclusters. Subsequently, these T cell subclusters were annotated using specific T cell subset markers [104,105], leading to the identification of 10 distinct T cell subsets. Similarly, to characterize the cellular subpopulations within monocyte subsets, the same methodological steps were repeated for monocyte types, followed by annotation using monocyte marker genes. Classical monocytes were identified based on CD14 expression, non-classical monocytes were defined by CD16 expression, and intermediate monocytes were characterized by the co-expression of both CD14 and CD16 [106].

### 4.4. Pseudo-Time Trajectories Analysis

Using the R package monocle (Version 2.28.0, https://bioconductor.org/packages/release/bioc/html/monocle.html, accessed on 5 August 2025) [107], we conducted a pseudotime analysis on T cell subsets derived from BM tissue samples of B-ALL cohorts to identify the changing trends of differential gene expression within these subsets. Differential genes among the T cell subsets were identified using the differentialGeneTest function. This was followed by dimensionality reduction and cell ordering based on DDRTree, focusing on genes that were significantly differentially expressed between the T cell subsets. Subsequently, the top 2000 genes with the most significant q-values were selected as ordering genes. We then analyzed the pseudotime-ordered cell data and specified nodes using the BEAM method from the monocle package. This analysis allowed us to display the expression trends of key genes across various states on the branch trajectory via a pseudotime heatmap. Additionally, we performed Gene Ontology (GO) functional enrichment analysis on these genes and visualized their expression at different time points using the plot_pseudo_time_heatmap function to generate a dynamic heatmap. It is noteworthy that pseudotime analysis constructs trajectories of cellular state changes based on overall gene expression similarity, with the aim of revealing continuous transcriptomic transitions. The results reflect the continuity of functional states and should not be interpreted as directly equivalent to strict lineage differentiation relationships.

### 4.5. Survival Analysis

Survival analysis for B-ALL cohorts was conducted using the R packages ‘survival’ (Version 3.5-7, available at https://github.com/therneau/survival, accessed on 6 August 2025) [108] and ‘survminer’ (Version 0.4.9, accessible at https://cran.rstudio.com/web/packages/survminer/index.html, accessed on 6 August 2025) [109]. The ssGSEA algorithm, implemented through the R package ‘GSVA’ (Version 1.46.0, found at https://www.bioconductor.org/packages/release/bioc/html/GSVA.html, accessed on 6 August 2025) [110], was utilized to quantify the B-ALL transcriptome data against the marker gene sets of ten T-cell subsets, thereby obtaining enrichment scores for each sample across all gene sets. Subsequently, the surv_cutpoint function from the survminer package was employed to ascertain the optimal cutoff value for the enrichment scores of each gene set. Based on this cutoff value, patients were classified into a ‘high-score group’ and a ‘low-score group,’ followed by the generation of Kaplan–Meier survival curves.

### 4.6. Metabolic Heterogeneity Analysis

To investigate the metabolic heterogeneity of T cell subsets, we employed the R package scMetabolism (Version 0.2.1, https://github.com/wu-yc/scMetabolism, accessed on 9 August 2025) [111] to evaluate the activities of metabolic pathways in T cells, which included a total of 85 Kyoto Encyclopedia of genes and genomes (KEGG) pathways. The AUCell algorithm was utilized to score each cell, resulting in the determination of activity scores for each cell across all metabolic pathways.

### 4.7. Consensus Clustering Analysis

Based on T-cell metabolic patterns, this study utilized the ConsensusClusterPlus R package (Version 1.62.0, https://github.com/renzhonglu/ConsensusClusterPlus, accessed on 9 August 2025) [112] to classify B-ALL cohorts into distinct groups. Initially, the average AUCell scores for each sample were computed based on 85 metabolic pathways. Subsequently, the optimal number of clusters (k) was determined by evaluating the stability of the cumulative distribution function (CDF) curve using the incremental area method. The incremental area measures the change in the area under the CDF curve between successive k values, reflecting improvements in clustering stability as the number of clusters increases. When the incremental area exhibited a significant decrease (indicating that additional clusters offered limited enhancement in stability), the elbow method was employed to identify the k value corresponding to the plateau of the incremental area (i.e., the inflection point where the marginal gain of adding new clusters diminishes). Ultimately, k = 4 was established as the optimal number of clusters. The clustering results were visually validated through a heatmap and PCA plots.

### 4.8. Cell–Cell Communication Analysis

To investigate the interactions between T cells and monocytes, we employed the R package CellChat (Version 1.6.1, https://github.com/jinworks/CellChat, accessed on 7 August 2025) [113] to analyze cell–cell communication among T cell subsets and monocytes. CellChat comprises a comprehensive database of human ligand-receptor interactions, facilitating the analysis of intercellular communication networks derived from single-cell sequencing data. We visualized the differences in communication pathway strength between T cell subsets and monocyte subsets using ggplot2 bar plots, and utilized the netVisual_bubble function to illustrate the specific signaling patterns of pathways within each subset.

### 4.9. Calculation of Transcription Factor Activity

To systematically assess TF activity, human regulon data, filtered for regulons with confidence levels A, B, and C, were obtained using the R package DoRothEA (Version 1.10.0, https://github.com/saezlab/DoRothEA, accessed on 7 August 2025) [114]. The VIPER method was employed to calculate TF activity scores, which were subsequently normalized using the scale method, with a minimum regulon size set to 4. The TF activity was integrated into the Seurat object, and clustering was performed through PCA dimensionality reduction, utilizing the first 10 principal components. Concurrently, T cell subset annotation information was incorporated into the Seurat object. For each subset, the top 20 TFs exhibiting the most significant differences across cell subsets were screened based on the VIPER activity scores of T cell subsets, and these were visualized through a heatmap, where color intensity reflects TF activity.

### 4.10. Machine Learning Algorithms

This study developed a machine learning model that integrates multiple algorithms to improve prediction accuracy. The training set was derived from the metabolic pathway scoring data of T cells across all B-ALL cohorts. The validation set was constructed by grouping T cell metabolic pathway scores by subtype, randomly dividing each group into several subsets, calculating the mean for each subset, sampling according to the proportion of cell numbers in each subtype, and finally integrating these to form a pseudo-bulk matrix for each T cell subtype.

The model evaluation encompasses 113 combinations of machine learning techniques. Independent prediction models encompass Ridge Regression and Support Vector Machine (SVM). The approach to enhancing predictive performance involves the sequential integration of glmBoost and Elastic Net (Enet) methodologies, which operate with a range of alpha values to provide flexibility during model building. In this context, Gradient Boosting Machine (GBM) plays a crucial role by correcting the errors that arise from the previous models, thus improving overall accuracy. Furthermore, Stepwise Regression (Stepglm) is utilized in conjunction with Ridge Regression, Enet, and Lasso models. This combination allows for a systematic variable selection process, which can be forward, backward, or bidirectional, aimed at optimizing the model’s predictive capability. In addition to these techniques, a variety of other models, including XGBoost, Linear Discriminant Analysis (LDA), Random Forest (RF), and Naive Bayes, are incorporated to maximize the unique benefits presented by various algorithms in specific scenarios.

### 4.11. Differential Gene Expression Analysis

Differentially expressed genes (DEGs) specific to the B-ALL groups were identified using the FindMarkers function from the R package Seurat. For the B-ALL single-cell dataset, the Wilcoxon rank-sum test was employed with the following thresholds: logfc.threshold = 0.25, min.pct = 0.25, and only.pos = TRUE, to identify highly expressed signature genes for each subtype. Genes that exhibited adjusted *p*-values below 0.05 after applying the Bonferroni correction were ultimately classified as DEGs.

### 4.12. Enrichment Analysis

The R package clusterProfiler (Version 4.6.2, https://bioconductor.org/packages/release/bioc/html/clusterProfiler.html, accessed on 2 August 2025) [115] was utilized to conduct functional enrichment analysis on the significantly DEGs identified through screening. This analysis included GO [116] and KEGG [117] to investigate the associated potential biological processes and pathways. The Benjamini–Hochberg method was employed for multiple test correction, and only the top five most significant GO functional annotation entries and the top five KEGG pathway analysis results were presented. Furthermore, drug enrichment analysis was performed using gene sets from the Drug–Gene Interaction Database (DGIdb) (https://dgidb.org/) [118] as a reference, with the Benjamini–Hochberg method also applied for multiple test correction to identify significantly enriched drugs with a *p*-value < 0.05.

### 4.13. Statistical Analysis

For data exhibiting non-normal distribution characteristics, this study employed the Wilcoxon rank-sum test for comparative analysis between two groups. For comparisons involving multiple groups, the Kruskal–Wallis rank-sum test was selected as the appropriate statistical method. A *p*-value greater than 0.05 was considered statistically non-significant, while a *p*-value of 0.05 or less was deemed statistically significant. The specific annotation rules for significance levels were as follows: * *p* ≤ 0.05, ** *p* ≤ 0.01, *** *p* ≤ 0.001, **** *p* ≤ 0.0001.

## 5. Conclusions

This study systematically elucidates the aberrant characteristics of the immune–metabolic network in B-ALL, characterized by a significant increase in Pro-B cells and a notable decrease in B cells, NK cells, monocytes, and pDCs. Different T cell subsets are significantly associated with the survival prognosis of B-ALL patients and exhibit heterogeneous metabolic patterns. Meanwhile, the intensity of the intercellular communication network demonstrates a significant degree of heterogeneity and is specific to the B-ALL groups, highlighting the complexity of immune regulation in B-ALL. Through an in-depth analysis of TF activity profiles and integration with drug enrichment screening data, multiple potential targets with individualized therapeutic value have been successfully identified. In the future, the application of these immunometabolic characteristics, core transcriptional regulatory factors, and candidate therapeutic drugs in clinical practice will not only enhance treatment strategies for B-ALL but also significantly improve clinical outcomes for patients, thereby demonstrating the clinical application value of personalized medicine in addressing disease-related metabolic disorders and other characteristics.

However, this study has several limitations and sources of variation that must be addressed in future research. These include residual technical variation remaining after batch correction when integrating multiple single-cell datasets, the heterogeneity associated with different genetic subtypes, ages, and risk stratifications of patients—which may significantly influence differences in metabolic subgroups—and intratumoral heterogeneity arising from subclonal structural differences in malignant B cells. Each of these factors may specifically impact the immune microenvironment. To enhance the clinical relevance of these findings, we propose a multi-tiered validation strategy. This strategy includes experimental validation of immune cell proportions and spatial interactions at the protein level through flow cytometry and spatial transcriptomics, functional validation and causal inference of T cell–monocyte crosstalk using an in vitro co-culture system combined with Seahorse metabolic analysis and pathway-blocking antibodies, and the application of machine learning classifiers to a large prospective cohort integrating genomic and clinical data. This rigorous evaluation will assess the associations between subtypes and genetic variations, recurrence risk, and response to immunotherapy, ultimately facilitating the translation of these findings into personalized treatment strategies.

## Data Availability

The datasets presented in this study can be found in online repositories. The names of the repository/s and accession number(s) can be found in the article/Supplementary Materials.

## Supplementary Materials

The following supporting information can be downloaded at: https://www.mdpi.com/article/10.3390/ijms26209996/s1.
