# Supplementary material for: Immunometabolic Dysregulation in B-Cell Acute Lymphoblastic Leukemia Revealed by Single-Cell RNA Sequencing: Perspectives on Subtypes and Potential Therapeutic Targets
Source: Int J Mol Sci. 2025 Oct 14;26(20):9996. doi: 10.3390/ijms26209996 (PMC12563021; doi:10.3390/ijms26209996)
Supplement: Supplementary file 1 [file ijms-26-09996-s001.zip › Supplementary Figure S2.pdf]

RF + NaiveBayes	1.000	0.747	0.979	0.871	1.000	0.919
RF + Enet[alpha=0.7]	0.977	0.937	0.851	0.699	0.999	0.893
RF + Enet[alpha=0.6]	0.977	0.924	0.828	0.732	1.000	0.892
Stepglm[backward] + plsRglm	0.792	0.895	0.919	0.883	0.972	0.892
Stepglm[both] + plsRglm	0.792	0.895	0.919	0.883	0.972	0.892
RF + Enet[alpha=0.8]	0.986	0.912	0.826	0.723	1.000	0.889
RF + Enet[alpha=0.1]	1.000	0.894	0.787	0.762	0.999	0.888
RF + Enet[alpha=0.5]	0.991	0.893	0.793	0.764	1.000	0.888
RF + glmBoost	0.982	0.923	0.845	0.687	1.000	0.887
RF + Enet[alpha=0.2]	1.000	0.890	0.786	0.754	0.999	0.886
RF + Enet[alpha=0.4]	1.000	0.885	0.794	0.751	1.000	0.886
RF + Enet[alpha=0.3]	1.000	0.889	0.790	0.750	0.999	0.886
RF + Enet[alpha=0.9]	0.964	0.945	0.871	0.639	0.994	0.883
Lasso + GBM	0.977	0.937	0.874	0.655	0.962	0.881
RF + GBM	1.000	0.895	0.838	0.672	0.989	0.879
RF + Lasso	0.955	0.950	0.879	0.598	0.989	0.874
Lasso + RF	0.982	0.922	0.867	0.640	0.957	0.874
Lasso + plsRglm	0.937	0.955	0.898	0.588	0.983	0.872
RF + Ridge	0.964	0.817	0.664	0.914	1.000	0.872
Lasso + NaiveBayes	0.941	0.948	0.886	0.591	0.988	0.871
glmBoost + GBM	0.991	0.903	0.854	0.634	0.972	0.871
Ridge	0.991	0.865	0.788	0.695	1.000	0.868
Enet[alpha=0.9]	0.950	0.931	0.861	0.615	0.982	0.868
RF + plsRglm	0.928	0.850	0.707	0.852	1.000	0.867
Enet[alpha=0.1]	1.000	0.862	0.778	0.682	1.000	0.864
GBM	1.000	0.878	0.833	0.618	0.993	0.864
Lasso + glmBoost	0.946	0.957	0.903	0.529	0.985	0.864
Lasso	0.937	0.922	0.848	0.624	0.973	0.861
Lasso + Stepglm[forward]	0.950	0.956	0.908	0.509	0.979	0.861
Enet[alpha=0.2]	1.000	0.852	0.772	0.677	1.000	0.86
Lasso + LDA	0.950	0.958	0.912	0.497	0.982	0.86
Lasso + Stepglm[backward]	0.955	0.950	0.911	0.529	0.948	0.859
Lasso + Stepglm[both]	0.955	0.950	0.911	0.529	0.948	0.859
NaiveBayes	0.995	0.815	0.975	0.505	1.000	0.858
Enet[alpha=0.8]	0.959	0.912	0.847	0.583	0.969	0.854
plsRglm	0.946	0.841	0.728	0.755	1.000	0.854
Stepglm[backward] + NaiveBayes	0.851	0.715	0.757	0.943	1.000	0.853
Stepglm[both] + NaiveBayes	0.851	0.715	0.757	0.943	1.000	0.853
Enet[alpha=0.6]	0.973	0.897	0.824	0.607	0.964	0.853
Enet[alpha=0.4]	0.991	0.853	0.778	0.638	0.994	0.851
Enet[alpha=0.5]	0.982	0.873	0.797	0.628	0.973	0.851
Enet[alpha=0.3]	1.000	0.841	0.764	0.647	1.000	0.85
RF	1.000	0.849	0.796	0.603	1.000	0.85
Enet[alpha=0.7]	0.991	0.878	0.811	0.587	0.959	0.845
glmBoost	0.986	0.888	0.825	0.577	0.946	0.845
glmBoost + plsRglm	0.946	0.893	0.857	0.545	0.958	0.84
glmBoost + NaiveBayes	0.977	0.849	0.830	0.547	0.995	0.839
glmBoost + RF	1.000	0.834	0.771	0.614	0.962	0.836
glmBoost + Lasso	1.000	0.848	0.802	0.541	0.958	0.83
glmBoost + Enet[alpha=0.9]	1.000	0.840	0.788	0.548	0.945	0.824
glmBoost + Enet[alpha=0.8]	1.000	0.819	0.770	0.527	0.948	0.813
glmBoost + Enet[alpha=0.7]	1.000	0.811	0.756	0.540	0.936	0.809
glmBoost + Enet[alpha=0.6]	1.000	0.808	0.752	0.554	0.928	0.808
glmBoost + Enet[alpha=0.5]	1.000	0.798	0.742	0.552	0.930	0.804
glmBoost + Enet[alpha=0.4]	1.000	0.796	0.736	0.562	0.924	0.804
glmBoost + Enet[alpha=0.3]	1.000	0.789	0.729	0.562	0.923	0.801
glmBoost + Enet[alpha=0.2]	1.000	0.784	0.727	0.564	0.922	0.799
glmBoost + Enet[alpha=0.1]	1.000	0.776	0.719	0.559	0.922	0.795
Stepglm[backward] + XGBoost	0.989	0.740	0.816	0.623	0.806	0.795
glmBoost + Ridge	1.000	0.770	0.713	0.557	0.921	0.792
XGBoost	1.000	0.740	0.655	0.657	0.903	0.791
LDA	1.000	0.720	0.603	0.671	0.883	0.775
Stepglm[backward] + glmBoost	0.928	0.738	0.686	0.538	0.980	0.774
Stepglm[both] + glmBoost	0.928	0.738	0.687	0.541	0.976	0.774
Stepglm[backward] + Ridge	0.946	0.716	0.678	0.556	0.948	0.769
Stepglm[both] + Ridge	0.946	0.716	0.678	0.556	0.948	0.769
Stepglm[both] + Lasso	0.986	0.708	0.711	0.519	0.887	0.762
Stepglm[backward] + Lasso	0.986	0.708	0.711	0.516	0.889	0.762
Stepglm[backward] + Enet[alpha=0.9]	0.986	0.707	0.707	0.512	0.888	0.76
Stepglm[backward] + Enet[alpha=0.3]	0.973	0.708	0.695	0.514	0.910	0.76
Stepglm[backward] + Enet[alpha=0.6]	0.973	0.709	0.699	0.506	0.911	0.76
Stepglm[backward] + Enet[alpha=0.8]	0.991	0.705	0.705	0.512	0.882	0.759
Stepglm[both] + Enet[alpha=0.8]	0.991	0.705	0.705	0.512	0.882	0.759
Stepglm[both] + Enet[alpha=0.6]	0.973	0.708	0.699	0.506	0.906	0.758
Stepglm[both] + Enet[alpha=0.4]	0.973	0.706	0.697	0.508	0.906	0.758
Stepglm[both] + Enet[alpha=0.7]	0.982	0.707	0.702	0.499	0.899	0.758
Stepglm[both] + Enet[alpha=0.9]	0.982	0.708	0.706	0.493	0.898	0.757
Stepglm[backward] + Enet[alpha=0.5]	0.982	0.706	0.700	0.502	0.897	0.757
Stepglm[backward] + Enet[alpha=0.7]	0.982	0.707	0.703	0.498	0.897	0.757
Stepglm[both] + Enet[alpha=0.2]	0.991	0.704	0.700	0.499	0.891	0.757
Stepglm[backward] + Enet[alpha=0.1]	0.973	0.705	0.696	0.509	0.902	0.757
Stepglm[both] + Enet[alpha=0.3]	0.982	0.705	0.700	0.503	0.894	0.757
Stepglm[backward] + Enet[alpha=0.2]	0.982	0.705	0.700	0.503	0.894	0.757
Stepglm[both] + Enet[alpha=0.1]	0.982	0.704	0.699	0.503	0.894	0.756
Stepglm[backward] + Enet[alpha=0.4]	0.986	0.704	0.701	0.498	0.892	0.756
glmBoost + LDA	1.000	0.698	0.637	0.543	0.902	0.756
Stepglm[both] + Enet[alpha=0.5]	0.991	0.704	0.702	0.494	0.886	0.755
Stepglm[backward] + LDA	0.977	0.678	0.658	0.542	0.919	0.755
Stepglm[both] + LDA	0.977	0.678	0.658	0.542	0.919	0.755
RF + XGBoost	1.000	0.615	0.584	0.599	0.952	0.75
glmBoost + XGBoost	0.934	0.744	0.752	0.553	0.691	0.735
Lasso + XGBoost	0.934	0.744	0.752	0.553	0.691	0.735
RF + SVM	1.000	0.562	0.536	0.526	1.000	0.725
SVM	1.000	0.562	0.536	0.526	1.000	0.725
Stepglm[both] + SVM	0.971	0.510	0.579	0.566	0.976	0.72
glmBoost + Stepglm[forward]	1.000	0.629	0.598	0.523	0.806	0.711
Stepglm[both] + RF	0.991	0.640	0.444	0.491	0.977	0.709
Stepglm[backward] + RF	0.991	0.624	0.462	0.487	0.971	0.707
glmBoost + Stepglm[backward]	1.000	0.562	0.528	0.500	0.924	0.703
glmBoost + Stepglm[both]	1.000	0.562	0.528	0.500	0.924	0.703
Stepglm[backward] + SVM	0.941	0.490	0.582	0.521	0.971	0.701
glmBoost + SVM	0.923	0.550	0.543	0.547	0.932	0.699
Stepglm[both] + GBM	0.959	0.623	0.402	0.637	0.857	0.696
Stepglm[backward] + GBM	0.950	0.631	0.399	0.628	0.865	0.695
Lasso + SVM	0.855	0.550	0.536	0.518	0.950	0.682
Stepglm[both] + XGBoost	0.896	0.683	0.654	0.629	0.534	0.679
RF + Stepglm[backward]	1.000	0.579	0.478	0.512	0.814	0.677
RF + Stepglm[both]	1.000	0.579	0.478	0.512	0.814	0.677
RF + LDA	1.000	0.520	0.521	0.542	0.764	0.669
Stepglm[backward]	1.000	0.524	0.534	0.468	0.813	0.668
Stepglm[both]	1.000	0.524	0.534	0.468	0.813	0.668
RF + Stepglm[forward]	1.000	0.541	0.489	0.584	0.602	0.643
Stepglm[forward]	1.000	0.522	0.510	0.460	0.544	0.607

0 0.5 1

AUC
1
0.8
0.6
0.4

Cohort
Cluster1
Cluster2
Cluster3
Cluster4
All T Cell
