# Supplementary figures and images for: Immunometabolic Dysregulation in B-Cell Acute Lymphoblastic Leukemia Revealed by Single-Cell RNA Sequencing: Perspectives on Subtypes and Potential Therapeutic Targets
Source: Int J Mol Sci. 2025 Oct 14;26(20):9996. doi: 10.3390/ijms26209996 (PMC12563021; doi:10.3390/ijms26209996)

# SingleR Annotation

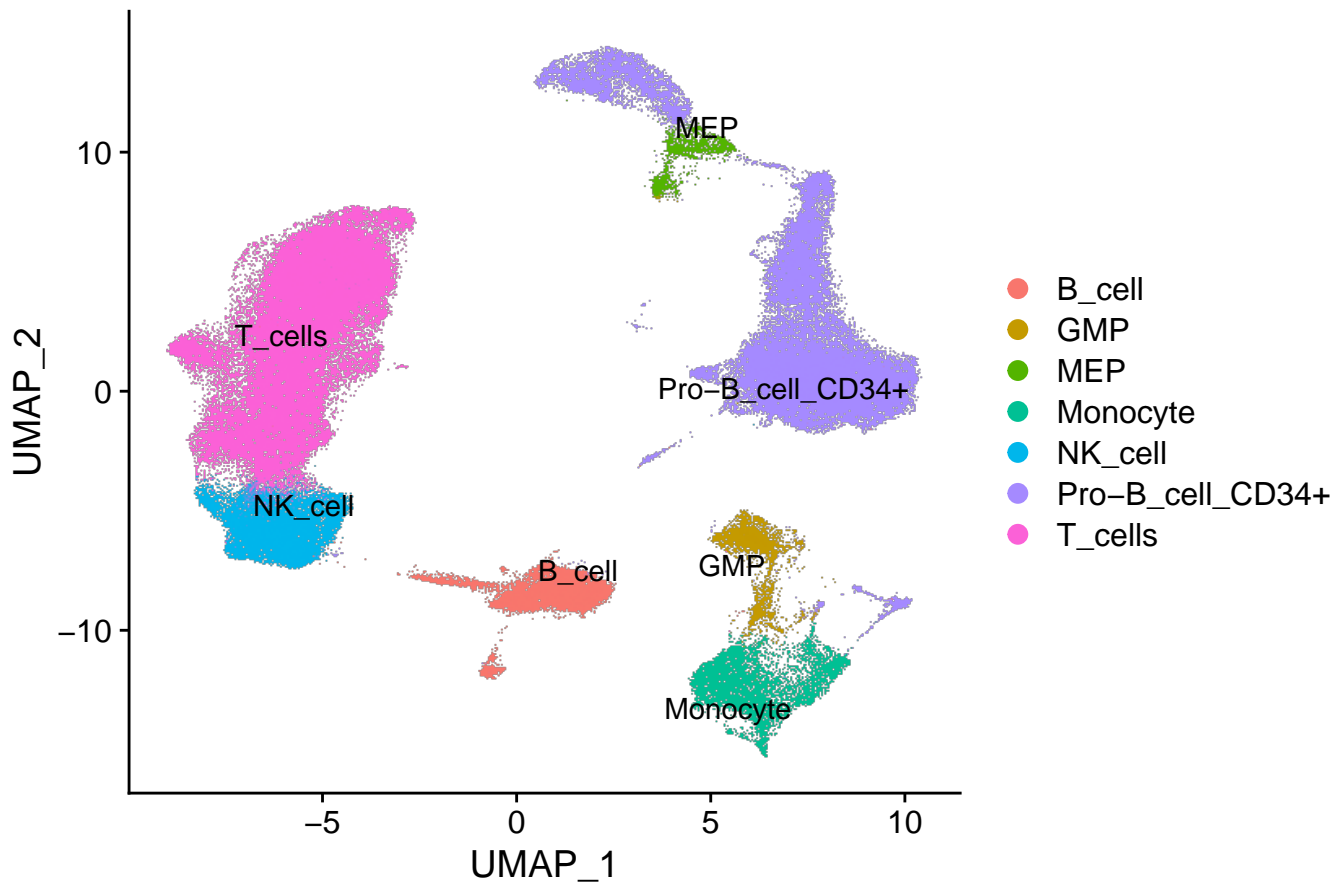

Supplement: Supplementary file 1 [file ijms-26-09996-s001.zip › Supplementary Figure S1.pdf]

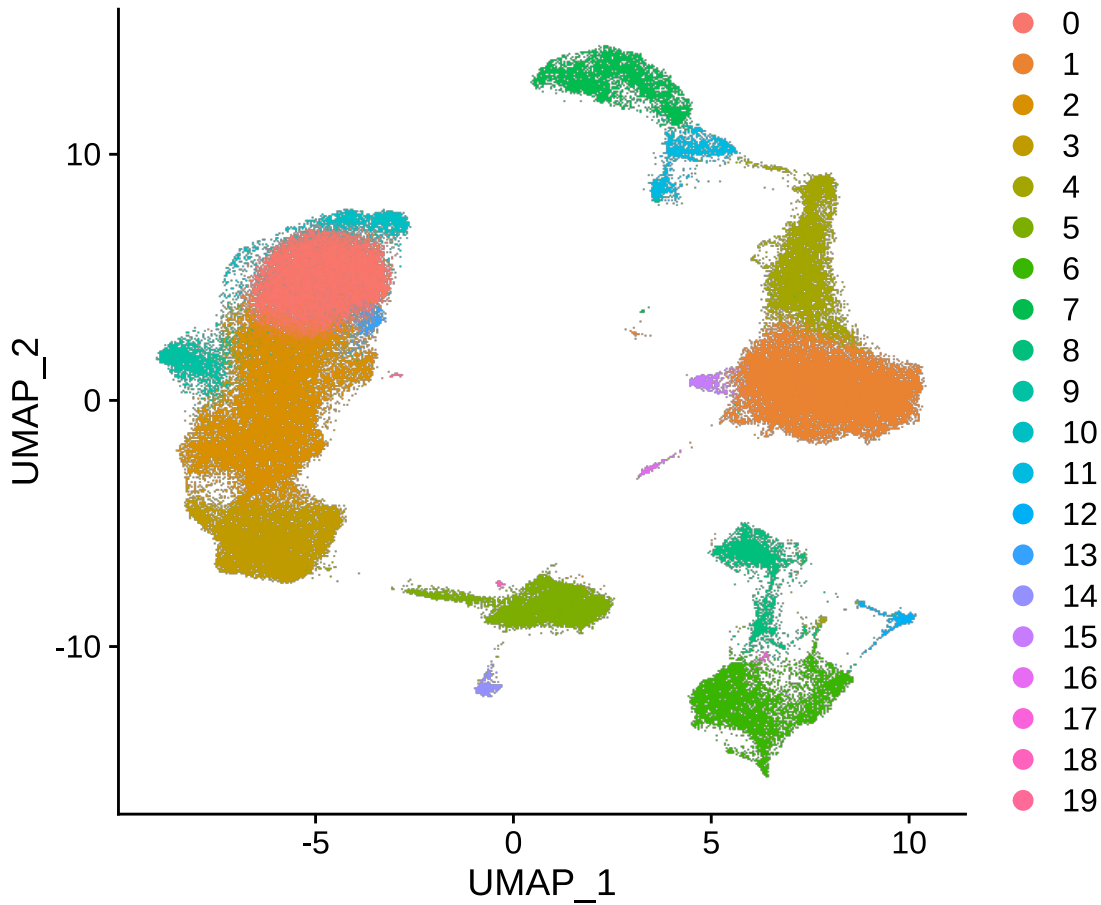

Supplement: Supplementary file 1 [file ijms-26-09996-s001.zip › Supplementary Figure S3.pdf]
